# Accurately deciphering spatial domains for spatially resolved transcriptomics with stCluster

**DOI:** 10.1093/bib/bbae329

**Published:** 2024-07-08

**Authors:** Tao Wang, Han Shu, Jialu Hu, Yongtian Wang, Jing Chen, Jiajie Peng, Xuequn Shang

**Affiliations:** School of Computer Science, Northwestern Polytechnical University, 1 Dongxiang Rd., Xi'an 710072, China; Key Laboratory of Big Data Storage and Management, Ministry of Industry and Information Technology, Northwestern Polytechnical University, 1 Dongxiang Rd., Xi'an 710072, China; School of Computer Science, Northwestern Polytechnical University, 1 Dongxiang Rd., Xi'an 710072, China; Key Laboratory of Big Data Storage and Management, Ministry of Industry and Information Technology, Northwestern Polytechnical University, 1 Dongxiang Rd., Xi'an 710072, China; School of Computer Science, Northwestern Polytechnical University, 1 Dongxiang Rd., Xi'an 710072, China; Key Laboratory of Big Data Storage and Management, Ministry of Industry and Information Technology, Northwestern Polytechnical University, 1 Dongxiang Rd., Xi'an 710072, China; School of Computer Science, Northwestern Polytechnical University, 1 Dongxiang Rd., Xi'an 710072, China; Key Laboratory of Big Data Storage and Management, Ministry of Industry and Information Technology, Northwestern Polytechnical University, 1 Dongxiang Rd., Xi'an 710072, China; School of Computer Science and Engineering, Xi'an University of Technology, No.5 South Jinhua rd., Xi'an 710048, China; School of Computer Science, Northwestern Polytechnical University, 1 Dongxiang Rd., Xi'an 710072, China; Key Laboratory of Big Data Storage and Management, Ministry of Industry and Information Technology, Northwestern Polytechnical University, 1 Dongxiang Rd., Xi'an 710072, China; School of Computer Science, Northwestern Polytechnical University, 1 Dongxiang Rd., Xi'an 710072, China; Key Laboratory of Big Data Storage and Management, Ministry of Industry and Information Technology, Northwestern Polytechnical University, 1 Dongxiang Rd., Xi'an 710072, China

**Keywords:** spatial transcriptomics, spatial domain identification, graph neural network, graph contrastive learning, multi-task learning

## Abstract

Spatial transcriptomics provides valuable insights into gene expression within the native tissue context, effectively merging molecular data with spatial information to uncover intricate cellular relationships and tissue organizations. In this context, deciphering cellular spatial domains becomes essential for revealing complex cellular dynamics and tissue structures. However, current methods encounter challenges in seamlessly integrating gene expression data with spatial information, resulting in less informative representations of spots and suboptimal accuracy in spatial domain identification. We introduce stCluster, a novel method that integrates graph contrastive learning with multi-task learning to refine informative representations for spatial transcriptomic data, consequently improving spatial domain identification. stCluster first leverages graph contrastive learning technology to obtain discriminative representations capable of recognizing spatially coherent patterns. Through jointly optimizing multiple tasks, stCluster further fine-tunes the representations to be able to capture complex relationships between gene expression and spatial organization. Benchmarked against six state-of-the-art methods, the experimental results reveal its proficiency in accurately identifying complex spatial domains across various datasets and platforms, spanning tissue, organ, and embryo levels. Moreover, stCluster can effectively denoise the spatial gene expression patterns and enhance the spatial trajectory inference. The source code of stCluster is freely available at https://github.com/hannshu/stCluster.

## Introduction

Many biological processes and disease mechanisms are intricately influenced by the spatial organization of cells within tissues [1]. Previous transcriptomics, although robust, offers only an averaged gene expression profile of bulk tissue, lacking essential spatial information about the localization of specific transcripts. Spatial transcriptomics (ST) preserves this critical inter-space context, providing insights into how cells function within their microenvironment, especially within heterogeneous tissues [2].

ST technologies integrate high-throughput RNA sequencing with histological imaging to map gene expression to precise locations within tissue, referred to as spots. Over time, the field of ST has witnessed notable improvements in sequencing resolution. While earlier technologies such as Spatial Transcriptomics [3] and 10x Visium [4] captured multiple cells per spot, recent advances such as Stereo-seq [5] and osmFISH [6] achieve single-cell or even subcellular resolutions. This significant enhancement allows researchers to delineate the spatial structure and function of tissues more accurately. Tissues typically exhibit a unique architectural organization crucial for their function [7]. Precisely identifying spatial domains enables the deciphering of underlying molecular mechanisms[8] governing tissue structure and function, thus proving pivotal in understanding the structural and functional organization of biological tissues. Additionally, it aids in mapping cell level micro-environment relationship and comprehending the interplay between different cell types, essential for processes such as cellular heterogeneity [9], multicellular mechanisms [10, 11], and oncology discoveries [12, 13].

The rationale behind spatial domain identification is to cluster spots together that exhibit similar expression patterns and spatial coherence. This clustering process is conducted primarily in an unsupervised manner, where patterns and relationships of spots are determined without utilizing prior domain-specific knowledge or labels. Due to the high-dimensional and high dropout rate characteristics of ST data, the key to accurate spatial domain identification lies in how to precisely learn the representation of spots based on the gene expression data and spatial information. In recent years, graph neural network (GNN) based methods have emerged as powerful techniques for identifying spatial domains in ST data. These GNN-based methods[14] have shown superior performance compared with spatially unaware clustering methods such as K-Means[15] and the Gaussian Mixture Model[16]. SEDR utilizes deep autoencoders to extract latent information from gene expression data and employs GNN to capture spatial information [17]. This combination enables the model to learn both gene expression patterns and spatial relationships simultaneously, facilitating improved understanding of cellular organization. SpaGCN incorporates histological information as an additional dimension alongside spatial data, enhancing the integration of neighboring cells’ gene expression profiles. By leveraging graph convolutional networks (GCNs), it improves cell representation for clustering tasks, leading to more accurate and meaningful grouping of cells based on their molecular characteristics [18]. STAGATE integrates spatial position into gene expression analysis using graph attention networks (GATs) [19], which allow for adaptive aggregation of neighboring cells’ gene expression information, resulting in more accurate cell clustering outcomes [20]. CCST leverages a deep graph infomax model [21] for representation learning by randomly constructing corrupted graphs and employing contrastive learning techniques. By optimizing model parameters through contrastive learning, CCST enhances the robustness of learned representations and improves the performance of downstream tasks such as cell clustering [22]. DeepST enhances feature vectors by integrating multiple sources of information, including histology images, gene expression similarity, and spatial proximity. By employing denoising autoencoders and Variational Graph Autoencoders, it improves the expressiveness of feature vectors, enabling more accurate characterization of cellular properties and relationships [23]. GraphST utilizes a contrastive learning approach and GNN to couple spatial positional information with gene expression data, enhancing the representation learning process. By effectively capturing spatial dependencies through contrastive learning, GraphST improves the quality of learned representations, leading to more informative and accurate cell representations [24]. The detailed model structure is concluded in Supplementary Table S1 and S2.

However, integrating gene expression with the spatial coordination information still faces challenges, resulting in less informative representations and lower domain identification accuracy. In this work, we introduce stCluster, a novel GNN-based deep learning framework, for representation learning and spatial domain identification for ST. stCluster is featured by its collaborative model optimization, which involves graph contrastive learning and multi-task learning. By incorporating graph contrastive learning, stCluster encourages the model to capture meaningful potential topology features while adaptively obtaining similar gene expression patterns from its adjacency neighbors. By jointly optimizing tasks, including gene expression reconstruction (GER), spatial adjacency graph (SAG) reconstruction, and deep embedding clustering (DEC), stCluster further fine-tunes the model to improve its ability to capture the complex relationships between gene expression and spatial organization. We systematically evaluate the performance of stCluster and compare it with state-of-the-art methods on cross-platform ST datasets. The results show that our method has superior performance in identifying complex spatial domains in tissue-level, organ-level, and embryo-level ST slices. Additionally, we also show that stCluster can be used to denoise the spatial gene expression patterns and enhance the spatial trajectory inference.

## Materials and methods

**Figure 1:**
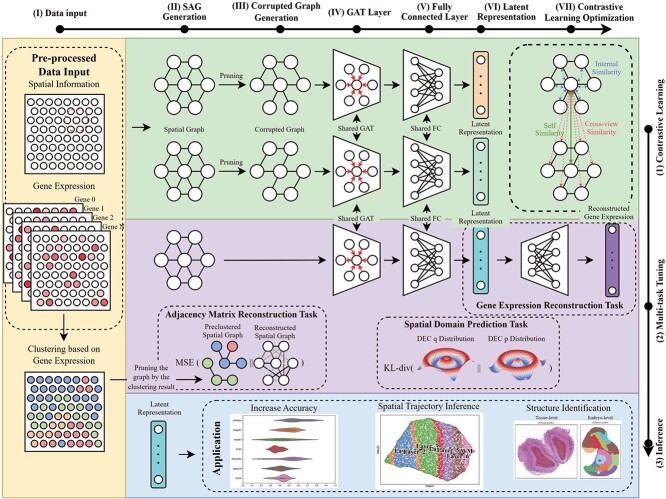
Overview of stCluster workflow. stCluster utilizes both gene expression and spatial information as inputs **(I)**, leveraging an attention-based graph encoder to derive latent representations **(II–VI)**. The model parameters are optimized through two approaches: graph contrastive learning **(1, VII)** and multi-task learning **(2)**. The resulting embeddings serve various downstream applications **(3)**, including the identification of spatial domains and biological structures, and inference of spatial trajectories.

In this study, we introduce stCluster, an innovative framework that utilizes deep GNNs to learn accurate representations of ST data and identify spatial domains. The stCluster framework consists of three main steps, as illustrated in Fig. 1. In the first step, the spatial gene expression profiles are encoded using a GAT-based graph encoder. This encoding process effectively captures the spatial dependencies and interactions among spots within the tissue. The second step of the stCluster framework involves a combined model optimization strategy that utilizes graph contrastive learning and multi-task learning. Contrastive learning enables the model to benefit from positive and negative spatial pairs, which enhances the discrimination capability of the generated representations. Multi-task learning, on the other hand, enables the model to simultaneously obtain multiple related tasks and improves the overall performance of the algorithm. By integrating these strategies, stCluster effectively learns the representation of each spot in the ST data. Finally, in the third step, stCluster applies a clustering algorithm to detect spatial domains based on the learned representations.

### Spatial graph construction

stCluster transforms spatial information into an SAG, with each spot as a node, and the edges are defined within a predefined radius (denoted as $r$). The radius value is obtained empirically and determines the maximum distance for edge connections. In our study, we fine-tuned the value of $r$ to achieve an average number of neighbors per spot ranging between 5 and 6. This parameter selection ensures that the graph captures meaningful spatial relationships while maintaining an appropriate level of connectivity for subsequent analyses. The generated adjacency matrix, denoted as $A$, represents the spatial relationships between spots. Specifically, if the Euclidean distance between a spot $i$ and a spot $j$ is less than the predefined radius $r$, an undirected edge is created between the two spots. This connection is represented in the adjacency matrix by setting the corresponding position to 1, otherwise 0.

### Graph encoder

To learn the latent representation of spots, stCluster takes a preprocessed gene expression matrix and the SAG $A$ as input. It utilizes a graph attention encoder consisting of two separate layers: the GAT [19] and a fully connected network layer.

The GAT layer plays a crucial role in capturing the spatial dependencies and interactions among spots. It employs attention mechanisms to assign different weights to the neighbors of each spot, thereby allowing the encoder to focus on the most informative features. Specifically, GAT first calculates the low-dimensional latent feature vector of the $i$-th spot’s feature vector $h_{i}$ by a linear transformation $W$. Secondly, GAT counts the aggregating weight for each spot pair by an attention vector $\vec{a}$ as follows: 


(1) \begin{align*} e_{ij} = \delta(\vec{a}^{T} (W_{GAT} h_{i} || W_{GAT} h_{j})),\end{align*}

where $e_{ij}$ is the attention score for the spot pair $(i, j)$, $\delta (\cdot )$ is the activation function, and $(\cdot ||\cdot )$ is the concatenation operator. Empirically, the Sigmoid function [25] has been selected as the activation function. $\vec{a}$ is a trainable attention vector shared for every spot in the graph, and $W_{GAT}$ is the trainable feature extraction matrix for GAT. To avoid excessive aggregating weight, GAT normalizes the aggregating weight for each spot as follows: 


(2) \begin{align*} \alpha_{ij} = softmax(e_{ij}) = \frac{\exp{(e_{ij})}}{\sum_{k \in N(i)} \exp{(e_{ik}})},\end{align*}

where $\alpha _{ij}$ is the attention weight for the spot pair $(i, j)$. Finally, like vanilla GNN methods, GAT aggregates the features of adjacency spots, and the central spot as follows: 


(3) \begin{align*} h_{i}^{(GAT)} = \delta\left(\sum_{j \in N(i)} \alpha_{ij} W_{GAT} h_{j}\right),\end{align*}

where $h_{i}^{(GAT)}$ is the latent representation of the spot $i$, $N(i)$ is the neighbor spot set of the spot $i$, and $\alpha _{ij}$ is the normalized aggregating weight.

For further extracting latent representations, we use another fully connected layer for the extra feature extraction process as follows: 


(4) \begin{align*} h_{i}^{(F)} = W_{F} \phi(h_{i}^{(GAT)}),\end{align*}

where $h_{i}^{(F)}$ is the latent representation of the spot $i$ learned by stCluster, $\phi $ is the ELU activation function [26], and $W_{F}$ is the trainable feature extraction matrix for fully connected layer. The output feature matrix of the fully connected layer $H^{(F)}$ is considered the output of the encoder and the latent representation matrix for spots learned by stCluster.

### Graph contrastive learning optimization

Inspired by GCA [27], stCluster employs graph contrastive learning as one of its optimization methods to optimize the model parameters. The contrastive learning optimization process comprises two primary steps: corrupted graph generation and contrastive optimization. In the corrupted graph generation phase, stCluster constructs two corrupted graphs by randomly pruning edges from the SAG using different pruning probabilities. The hyperparameter $c_{k}$$(k\in \{1,2\})$ controls the pruning probabilities for the two corrupted graphs and is set to default values of 0.05 and 0.1, respectively. The edge pruning probability of the spot pair $(i, j)$, denoted as $c_{ij}$, is defined as follows:


(5) \begin{align*} c_{ij} = min\left(\frac{\alpha_{max} - \alpha_{ij}}{\alpha_{max} - \alpha_{mean}} \cdot c_{k}, \gamma\right)\end{align*}

In this context, $\alpha _{ij}$ represents the cosine similarity between the spot pair $(i, j)$, while $\alpha _{max}$ and $\alpha _{mean}$ denote the maximum and average cosine similarity values among all spot pairs. Additionally, $\gamma $ is the truncation probability that serves to limit the pruning rate, with stCluster employing a default value of 0.7. In accordance with the diverse pruning probabilities, stCluster generates two corrupted graphs, referred to as view $U$ and view $V$.

Following that, stCluster receives the gene expression data along with the two corrupted graphs as inputs and proceeds to train the embedding using the graph encoder independently for each view. The embedding vectors of a spot $i$ in the two views, denoted as $(u_{i}, v_{i})$, are considered as self pairs. Pairs consisting of the spot $i$ and other spots within the same view, such as $(u_{i}, u_{k})$, are treated as internal pairs. Similarly, pairs including the spot $i$ and spots from the other view, such as $(u_{i}, v_{k})$, are regarded as cross-view pairs. In the context of graph contrastive learning, the optimization objective is to enhance the distinguishability of each spot’s embedding from the embeddings of other spots. Essentially, this involves maximizing the similarity of self pairs (self-similarity) while minimizing the similarities of internal pairs (internal similarity) and cross-view pairs (cross-view similarity). To summarize, the loss function for the spot $i$ is formulated as follows: 


(6) \begin{align*} l(u_{i}) = \log\left(\frac{e^{(u_{i} \cdot v_{i}) / \tau}}{e^{(u_{i} \cdot v_{i}) / \tau} + \sum_{k \neq i} e^{(u_{i} \cdot v_{k}) / \tau} + \sum_{k \neq i} e^{(u_{i} \cdot u_{k}) / \tau}}\right),\end{align*}

where $l(u_{i})$ is the loss of the spot $i$ in view $U$, $u_{i}$ and $v_{i}$ are the embedding vectors of the spot $i$ in view $U$ and view $V$, respectively. $\tau $ is a hyperparameter and is set to 0.5 by default. To compute the overall loss function, the sum of all spots’ losses in both views is averaged. Let us suppose the number of spots is represented by $N$. Hence, the overall loss function can be expressed as


(7) \begin{align*} L_{CL} = \frac{1}{2N} \sum_{i=1}^{N} (l(u_{i}) + l(v_{i}))\end{align*}

### Multi-task learning optimization

To further optimize model parameters, stCluster employs multi-task learning optimization which covers three tasks: adjacency matrix reconstruction (AMR), GER, and spatial domain prediction (SDP).

**Adjacency matrix reconstruction task.** The objective of the AMR task is to reconstruct the adjacency matrix $A^{(R)}$, leveraging the learned embeddings $H^{(F)}$ through inner product operations. The goal is to make $A^{(R)}$ as similar as possible to the cell-type aware spatial adjacency graph (ctSAG), denoted as $A^{(P)}$. To generate the ctSAG, it first utilizes the k-nearest neighbors algorithm [28] to generate a denser graph network $A^{(P0)}$. For instance, we set $k$ as 35 to ensure that each spot has 35 neighboring spots. Subsequently, the Louvain clustering algorithm [29] is applied to derive an initial clustering result denoted as $C$, based on the gene expression profiles. In order to construct the ctSAG, for each edge in $A^{(P0)}$, if its two end spots belong to different clusters, the edge will be pruned with a probability $\theta $, setting as 0.96 by default. The goal is to optimize $A^{(R)}$ to closely resemble $A^{(P)}$ by optimizing the mean square error (MSE) loss as follows:


(8) \begin{align*} l_{adj} = MSE(A^{(P)}, A^{(R)})\end{align*}

**Gene expression reconstruction task.** stCluster minimizes the MSE loss to reduce the discrepancy between the original gene expression ($H$) and the reconstructed gene expression ($\phi (W_{gene} H^{(F)}$)), where $\phi $ represents the ELU activation function. The trainable linear weight for the GER task is denoted as $W_{gene}$, and $H^{(F)}$ represents the latent representation learned by the graph encoder. The MSE loss is calculated as follows: 


(9) \begin{align*} l_{gene} = MSE(H, \phi(W_{gene} H^{(F)}))\end{align*}

**Spatial domain prediction task.** In the SDP task, stCluster applies the DEC algorithm [30] to refine the clustering performance based on the learned representation through an iterative, joint optimization process. The learned latent representations are used to generate a soft clustering distribution $Q$ based on t-distribution [31] as follows: 


(10) \begin{align*} q_{ij} = \frac{(1 + ||h^{(F)}_{i} - \mu_{j}||^{2})^{-1}}{\sum_{k=1}^{K} (1 + ||h^{(F)}_{i} - \mu_{k}||^{2})^{-1}},\end{align*}

where $q_{ij}$ is the probability that a spot $i$ belongs to the $j$-th cluster, $h^{(F)}_{i}$ is the latent representation of the spot $i$, and $\mu _{j}$ is the centroid vector of the $j$-th cluster. The initial centroid is obtained by averaging the nodes’ representations in the cluster after the K-Means clustering [15]. And $K$ is the cluster number. Next, stCluster corrects the distribution $Q$ by the auxiliary distribution $P$ as follows: 


(11) \begin{align*} p_{ij} = \frac{q_{ij}^{2} / \sum_{i=1}^{N} q_{ij}}{\sum_{k=1}^{K} (q_{ik}^{2} / \sum_{i=1}^{N} q_{ik})},\end{align*}

where $p_{ij}$ is the auxiliary probability for $q_{ij}$, $N$ is the number of neighbor spots of the spot $i$. Finally, stCluster minimizes the KL divergence [32] of distribution $Q$ and auxiliary distribution $P$ as follows:


(12) \begin{align*} l_{pred} = KL(P||Q) = \sum_{i}\sum_{j} p_{ij} \log \frac{p_{ij}}{q_{ij}}\end{align*}

In summary, the multi-task optimization framework involves the combination of three optimization tasks in the following manner: 


(13) \begin{align*} L_{MT} = \alpha_{adj} \cdot l_{adj} + \alpha_{gene} \cdot l_{gene} + \alpha_{pred} \cdot l_{pred},\end{align*}

where $L_{MT}$ is the loss of multi-task optimization, and $\alpha _{adj}$, $\alpha _{gene}$, and $\alpha _{pred}$ are the weights for each task. The details of the evaluation of the model hyperparameters can be find at Supplementary Note 1 and Supplementary Figure S1-1.

### Collaborative optimization of graph contrastive learning and multi-task learning

During the training process, stCluster employs an intermittently collaborative optimization strategy. It applies graph contrastive learning optimization in each iteration and utilizes multi-task optimization to fine-tune the model parameters periodically. By default, stCluster sets the interval for fine-tuning to 50 iterations. The final loss function can be expressed as follows: 


(14) \begin{align*} \begin{split} L_{final} = \left \{ \begin{array}{ll} L_{CL}, & epoch\ \% \ inv \neq 0 \\ L_{CL} + L_{MT}, & epoch\ \% \ inv = 0\\ \end{array}, \right. \end{split}\end{align*}

where $L_{final}$ is the final loss function for each epoch, $\%$ is the remainder operation, and $inv$ is the interval hyperparameter.

### Clustering

To generate the clustering result, we feed the representations learned by stCluster into the Mclust clustering algorithm [33] to identify spatial domains. Mclust is a widely utilized clustering package in R that models data using a Gaussian finite mixture approach, offering a variety of covariance structures and the option to select different numbers of mixture components. Specifically, stCluster employs Mclust version 5.4.10 with the ”EEE” method to cluster the latent representations due to the better clustering performance (Supplementary Figure S1-2). For other spatial clustering methods included in our comparison, we used the default clustering methods specified by each respective method (as detailed in Supplementary Table S2).

Importantly, while the representation learning approach employed by stCluster does not require prior knowledge of the number of domains, using the Mclust algorithm to generate clustering results necessitates specifying the number of clusters. For datasets where the number of clusters is unknown, we recommend using the Louvain community detection algorithm [29] to derive clustering results. This method is particularly advantageous in scenarios where the cluster count is not predetermined.

Additionally, we have conducted an experiment to assess stCluster’s ability to identify domains in unlabeled datasets. The results of this evaluation can be found in the Supplementary Information, Supplementary Figure S1-3.

### Benchmarking

The representation learning and spatial domain detection capabilities of stCluster are compared with six state-of-the-art methods: SEDR [17], SpaGCN [18], STAGATE [20], CCST [22], DeepST [23], and GraphST [24]. For more detailed information on these compared methods, please refer to Supplementary Table S2. The evaluation of clustering results is performed on multiple datasets using the adjusted rand index (ARI) [34], the normalized mutual information (NMI) [35], and the Intersection over Union (IoU) metrics [36]. The ARI and NNI quantify the effectiveness of clustering by counting the number of true label-predicted label pairs assigned to the same or different clusters. The IoU assesses the overlap between the ground truth region and the corresponding predicted label region. Specifically, we use the Hungarian algorithm [37] to match the predicted cluster labels with the ground truth labels in this scenario. Details of the three metrics can be found in Supplementary Information.

### Data availability

Briefly, we utilized stCluster on ST datasets generated by various technologies, including Spatial Transcriptomics [3], 10x Visium [4], Stereo-seq [5], and osmFISH [6]. Specifically, the dorsolateral prefrontal cortex (DLPFC) dataset [38] comprises 12 slices of human DLPFC samples. The number of spots ranges from 3498 to 4789 for each slice, and manual annotations are provided by the authors of the dataset. We also evaluated four slices of mouse brain obtained from the 10x Genomics database (https://www.10xgenomics.com/resources/datasets), with spot numbers ranging from 2696 to 3353. The Mouse Organogenesis Spatiotemporal Transcriptomic Atlas [39] and Zebrafish Embryogenesis Spatiotemporal Transcriptomic Atlas [40] were obtained using the Stereo-seq technique, with spot numbers ranging from 4356 to 5913 and 13 166 spots, respectively. In addition, we utilized mouse olfactory bulb data obtained through ST, 10x Visium, and Stereo-seq techniques, which consist of 264, 918, and 10 000 spots, respectively. Furthermore, we employed a mouse somatosensory cortex dataset obtained via the osmFISH technique, comprising 5328 spots. Datasets without explicit sources mentioned were obtained from the STomicsDB database (https://db.cngb.org/stomics/). Supplementary Table S3 summarizes the detailed information of seven datasets used in this work. A detailed tutorial of stCluster is available at https://stcluster.readthedocs.io/en/latest/.

### Data preprocessing

In all datasets, we initially remove spots located outside the main tissue area. As a standardized procedure, for datasets with over 3000 genes per spot, we select the top 3000 variable genes (HVGs) from the original gene expression profiles for downstream analysis. Subsequently, the raw gene expressions are log-transformed and scale-normalized using the Python package SCANPY [41]. We also evaluate the influence of the different numbers of the input HVGs in Supplementary Note 1 and Supplementary Figure S1-4.

### Visualization and trajectory inference

The box plots and violin plots in this article are drawn by the Python package matplotlib. The UMAP visualization plots [42] and PAGA plots [43] for trajectory inference are generated by the Python package SCANPY [41].

## Results and discussion

### Overview of stCluster workflow

In this work, we present stCluster, a deep GNN framework tailored for representation learning and domain identification for spatially resolved transcriptomics (Fig. 1). This novel framework integrates spatial topology with spot-level gene expression through a GAT-based encoder. To learn better low-dimensional embedding for each spot, stCluster synergistically combines graph contrastive learning and multi-task learning in the model optimization, both of which are cutting-edge deep learning techniques.

Our analysis compared the spatial domain identification accuracy of stCluster with six other state-of-the-art techniques, namely SEDR [17], SpaGCN [18], STAGATE [20], CCST [22], DeepST [23], and GraphST [24]. Notably, existing methods mainly utilize the GCNs for aggregating spot information from neighboring spots. In our method, besides using the GNN, stCluster also adopts a contrastive learning strategy within the auto-encoder structure. This allows for a more comprehensive learning of topology features within spots. Additionally, stCluster employs a multi-task learning strategy to fine-tune model parameters, enabling better capture of complex biological features inherent in spots. We thoroughly compared existing frameworks with stCluster in a step-by-step manner, characterizing main steps of algorithms and their similarities/dissimilarities, which are detailed in the Supplementary Note 2.

stCluster treats the pre-processed gene expression and the spatial coordination information as input (Fig. 1 (I)). During the training stage, stCluster first constructs the SAG (Fig. 1 (II)) based on the spatial information of spots. Subsequently, stCluster randomly prunes the SAG into two different corrupted graphs (Fig. 1 (III)) and learns representations (Fig. 1 (VI)) for each spot by a GAT layer (Fig. 1 (IV)) and a fully connected layer (Fig. 1 (V)) based on these two different SAGs. The model parameters are then optimized using contrastive learning optimization (Fig. 1 (VII, 1)). Every 50 epochs, stCluster utilizes the multi-task optimization to further tune the model parameters optimized through contrastive learning (Fig. 1 (2)). The representations utilized in this multi-task optimization process are obtained from the original SAG. The ultimate objective of these optimization strategies is to integrate the gene expression data more effectively with the spatial information embedded within the spots.

During the inference stage, stCluster generates representations using the original SAG and employs the Mclust clustering algorithm [33] to identify spatial domains based on latent representations (Fig. 1 (3)).

### stCluster enhances the accuracy of domain detection within the human DLPFC

To assess the capability in recognizing spatial domains, we applied stCluster to 12 slices of the human DLPFC that were sequenced using the 10x Visium technology [38]. The DLPFC dataset has been manually annotated to delineate six cortical layers and white matter, serving as the ground truth for evaluation. Our analysis compared the spatial domain identification accuracy of stCluster with six other state-of-the-art techniques, namely SEDR [17], SpaGCN [18], STAGATE [20], CCST [22], DeepST [23], and GraphST [24]. For the six compared methods, we used their default parameter settings to learn the latent representations. We then applied their default clustering methods to generate spatial domains for comparison (see Supplementary Table S2 for more details). The ARI [34], the NMI [35], and the IoU[36] were used as the metrics to assess the clustering performance of each method.

**Figure 2:**
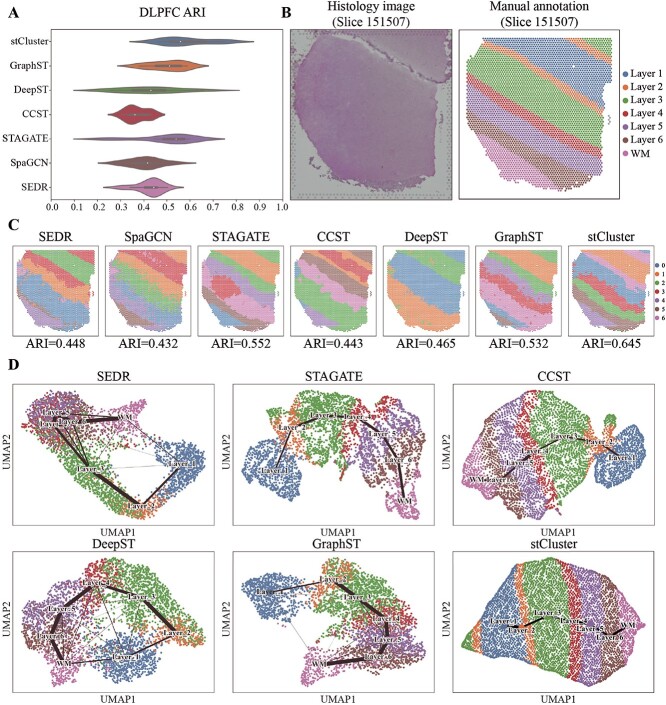
stCluster effectively improves the accuracy of spatial domain detection in the human DLPFC spatial transcriptome dataset. **(A)** The violin plot of the ARI scores of seven clustering methods in 12 DLPFC slices. In the violin plot, the width of the ”violin” represents the data density; the center white point, box limits, and whiskers denote the median, upper and lower quartiles, and 1.5x interquartile range, respectively. **(B)** The histology image and manual annotations of cortex layers and white matter (WM) in the DLPFC slice 151507. **(C)** Visualization of detected spatial domains by SEDR, SpaGCN, STAGATE, CCST, DeepST, GraphST, and stCluster in DLPFC slice 151507. **(D)** UMAP visualizations paired with PAGA plots are produced based on the representations from SEDR, STAGATE, CCST, DeepST, GraphST, and stCluster. The PAGA plots are superimposed onto the UMAP plots. Notably, SpaGCN does not produce latent representations, rendering it incompatible with UMAP visualization and subsequent PAGA plotting.

Figure 2A shows the distribution of ARI scores across 12 DLPFC slices obtained by stCluster and six compared methods. It is shown that stCluster improves the accuracy in identifying the hierarchical structure of the cortex layers, with an average ARI of 0.58, and the best ARI of 0.75, while GraphST and STAGATE achieve average ARI scores of 0.5 and 0.49, respectively. We visualized the slice 151507 for an illustration (Fig. 2B and C); stCluster can identify all domains with proper boundaries and achieves the best accuracy compared with other methods. SEDR, SpaGCN, and GraphST show a mixing of two layers within certain local regions at the boundaries of layers 4, 5, and 6. Although STAGATE, CCST, and DeepST can clearly depict the boundaries between different layers, they display an incorrect layer shape in layers 4 to 6. The NMI and IoU scores also fit the trend of the ARI score across the 12 slices which further proves the effectiveness of stCluster. The full summary of performance on the 12 slices can be found in Supplementary Table S4, S5, and S6 and Supplementary Figure S1.

Effective latent representations should accurately mirror the original relative positions of spots and spatial domains. To this end, we visualized the latent representations of the same DLPFC slice obtained from different methods using a dimensionality-reduced UMAP plot. As shown in Fig. 2D, the representations learned by stCluster accurately restore the spot distribution and the orders of spatial domains. SEDR fails to differentiate among layers 4, 5, and 6, whereas DeepST faces challenges in effectively distinguishing between the white matter and the layer 1. While STAGATE, GraphST, and CCST manage to represent the domain-level locations, they fall short in accurately depicting the spot-level positions. Notably, stCluster stands alone in its ability to restore both instances of the layer 2 and the layer 3. Furthermore, we utilized the PAGA algorithm [43] to validate the trajectories inferred from the latent representations. The PAGA plots were overlaid on the UMAP plots as displayed in Fig. 2D. It is evident that the PAGA plots derived from stCluster, STAGATE, and CCST accurately depict layers 1 through 6 and white matter with linear developmental trajectories. Conversely, the trajectories from SEDR, DeepST, and GraphST form circular paths that diverge from manual annotations.

### stCluster consistently performs well across various spatial resolutions and sequencing platforms

The evolution of spatial transcriptomic technologies has led to varying spatial resolutions based on the choice of sequencing platforms. To test stCluster’s adaptability across these platforms, we performed spatial domain identification across four platforms with various resolutions: Stereo-seq [5], 10x Visium [4], Spatial Transcriptomicss [3], and osmFISH [6].

**Figure 3:**
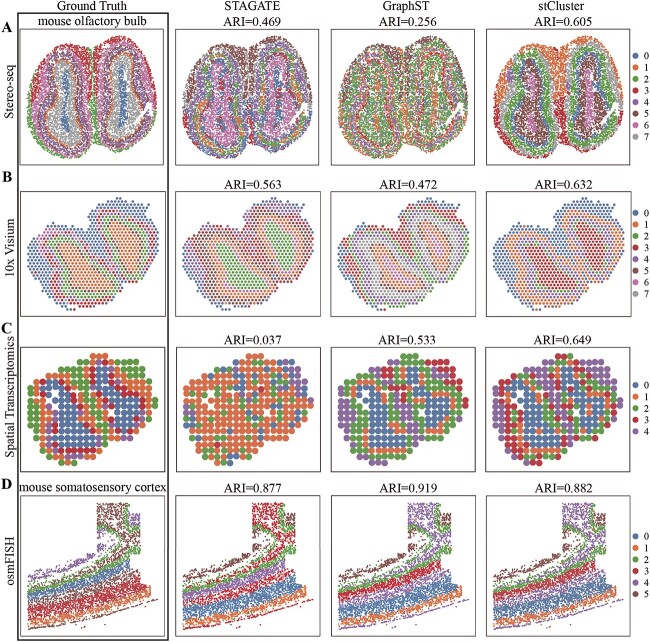
stCluster enables discriminating cell types in the datasets obtained by diverse spatial resolutions and methods. The manual annotation and domain detection results for STAGATE, GraphST, and stCluster for the Stereo-seq mouse olfactory bulb dataset **(A)**, 10x Visium mouse olfactory bulb dataset **(B)**, ST mouse olfactory bulb dataset **(C)**, and osmFISH mouse somatosensory cortex dataset **(D)**.

Our evaluation first employed ST data from the mouse olfactory bulb tissue sequenced by Stereo-seq, 10x Visium, and Spatial Transcriptomic. Specifically, Stereo-seq attains a single-cell spatial resolution with a 220 nm diameter per spot. In contrast, 10x Visium offers a spatial resolution of 55 $\mu $m per spot, covering multiple cells, while Spatial Transcriptomics provides a coarser spatial resolution at 100 $\mu $m per spot. The Stereo-seq dataset contains 10 000 spots, the 10x Visium dataset has 918 spots, and the Spatial Transcriptomics dataset comprises 264 spots. Figures 3A, B, and C display the ground truth structure of mouse olfactory bulb, alongside the clustering results of stCluster and its top two competitors, STAGATE and GraphST. Evidently, stCluster consistently outperforms across varying resolutions and platforms. Furthermore, stCluster showcases the ability to identify and differentiate the complex spatial structures intrinsic to the various layers of the olfactory bulb.

We further assessed our approach using data from the mouse somatosensory cortex sequenced via osmFish, a platform characterized by its sub-cellular spatial resolution [6, 44]. Unlike the previously mentioned platforms, osmFish quantifies expression levels of specific transcripts within tissue sections but for a more limited gene set. This dataset comprises 5328 spots, with measurements for only 33 genes per spot. Figure 3D depicts the layer identification performance. Notably, all three methods exhibit high accuracy, with stCluster ranking second. This heightened accuracy could be attributed to the dataset’s focus on genes that serve as markers for individual layers.

### stCluster facilitates accurate identification of tissue structures in mouse brain

**Figure 4:**
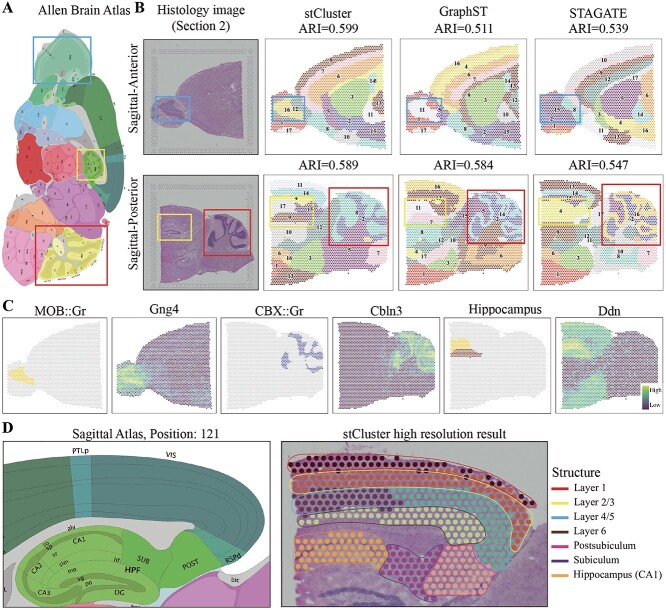
stCluster accurately distinguishes different structures in the mouse brain. **(A)** The Allen brain atlas of the sagittal mouse brain in position 121. **(B)** The histology image, spatial domain identification of stCluster, GraphST, and STAGATE of the anterior slice (up row) and posterior slice (down row), respectively. **(C)** The spatial distribution of structures the Main olfactory bulb-granule layer(MOB::Gr), the Hippocampus, and the Cerebellar cortex-granular layer(CBX::Gr) stCluster predicted and the expression levels of corresponding marker genes Gng4, Cbln3, and Ddn, respectively. **(D)** isocortex and hippocampal formation areas annotated by the Allen brain atlas in position 121 (left), and the finer-grained clustering result of stCluster and the manual annotation (right).

To assess stCluster’s ability to decipher the complex tissue structures at the organ level, we used two sections of the 10X Visium ST data of the mouse brain from 10x Genomics database. Each section was separated into anterior sagittal and posterior sagittal portions, as depicted in Fig. 4A. We used stCluster, GraphST, and STAGATE to identify the tissue structures. Figure 4B visualizes the clustering results of one section compared with the histology image. The ARI scores were calculated based on the spot labels from the StomicsDB database. Notably, stCluster consistently outperforms with the highest ARI scores across both slices. Furthermore, stCluster adeptly identifies the granule layer (highlighted by a blue frame in Fig. 4B) in the anterior slices, and both the hippocampal region (outlined in yellow in Fig. 4B) and the cerebellar cortex (encased in red in Fig. 4B) in the posterior slices. These identified structures align well with the histology image and the Allen brain map (as shown in Fig. 4A). While STAGATE and GraphST are capable of discerning these regions, they manifest a diminished overall accuracy and an overextended granule layer region. The results from the alternate section 2, which also mirror these findings, are detailed in Supplementary Figure S2C.

Additionally, the spatial distribution of marker gene expressions corroborates the brain tissue structures pinpointed by stCluster (see Fig. 4C). For instance, the protein-coding gene *Gng4* shows pronounced expression within the granule layer of the mouse’s olfactory area. In contrast, *Ddn* presents a discernible expression pattern, differentiating the hippocampal formation from other regions; it is particularly upregulated in the Ammon’s horn and Dentate gyrus regions. Another gene, *Cbln3*—recognized as the third member of the precerebellin family [45]—is markedly expressed in the granular layer of the cerebellar cortex. These expression patterns of marker genes harmoniously map onto the structures we identified. Comparable observations in the other section are delineated in Supplementary Figure S2D.

stCluster also has the ability to discern fine-grained structures by increasing the value of the cluster setting parameter in Mclust, leading to a greater number of clusters. As depicted in Fig. 4D, this allows stCluster to illuminate the intricate structure of the isocortex-hippocampal region as annotated by the Allen brain atlas. stCluster can precisely distinguish layers within the visual areas of the isocortex. Moreover, it identifies the detailed structural distinctions within the hippocampal CA1 field and the retrohippocampal region. The retrohippocampal region can be further clustered into post-subiculum and subiculum structures with notable accuracy. Parallel detection of these fine-grained structures in the other section is illustrated in Supplementary Figure S2E.

### stCluster effectively identifies tissue structures in mouse embryos and zebrafish

Another crucial application of spatial transcriptomic technologies lies in studying embryonic development. The spatial distribution of transcripts in embryos provides a molecular map that guides the formation and differentiation of tissues and organs. Different from ST studies with previous tissue-level sections, the embryo-level cellular landscape is highly heterogeneous with various cell types emerging and differentiating simultaneously [39, 40]. In this section, we evaluated the performance of stCluster with two embryo datasets: Mouse Organogenesis Spatiotemporal Transcriptomic Atlas (MOSTA) [39] and Zebrafish Embryogenesis Spatiotemporal Transcriptomic Atlas (ZESTA) [40], both of which were acquired using Stereo-seq.

**Figure 5:**
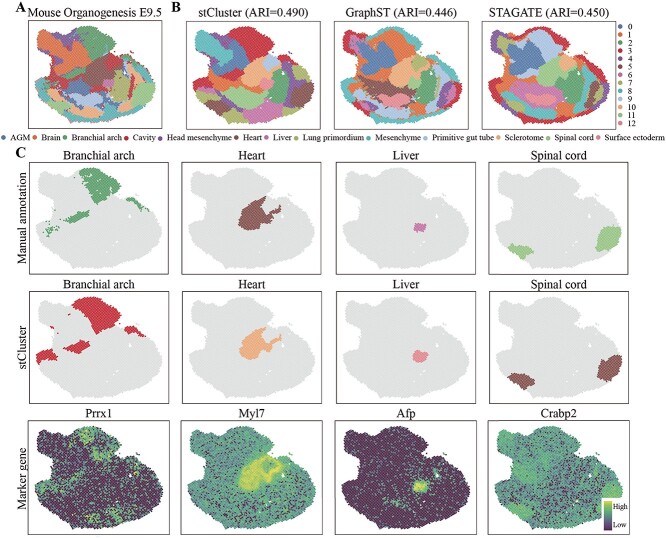
stCluster effectively improves the identification of known tissue structures in the mouse embryo (MOSTA dataset). **(A)** The manual annotation of the MOSTA E9.5 E2S3 slice. **(B)** The clustering result of stCluster, GraphST, and STAGATE at the MOSTA E9.5 E2S3 slice. **(C)** Row 1: The manual annotation of the spatial distribution of the branchial arch, heart, liver, and spinal cord; Row 2: The stCluster identified domain’s spatial distribution of the branchial arch, heart, liver, and spinal cord. Row 3: The expression levels of marker genes of the branchial arch, heart, liver, and spinal cord.

We assessed the expression of marker genes in various organ tissues identified by stCluster using the mouse embryo data from the MOSTA dataset [39]. Figure 5A represents the manually annotated E2S3 slice, sectioned from the embryo at embryonic day 9.5 (E9.5), which encompasses 5059 cells distributed across 13 distinct organ tissues. In spatial domain identification, stCluster outperforms both GraphST and STAGATE, as evidenced in Fig. 5B. Figure 5C illustrates the domains of the branchial arch, heart, liver, and spinal cord as identified by stCluster. Additionally, it displays the expression levels of four marker genes: *Prrx1, Myl7, Afp*, and *Crabp2* corresponding to these organs. Clearly, the domains delineated by stCluster align closely with manual annotations and regions of heightened gene expression. Further illustrations of four other mouse embryos are available in Supplementary Figure S5. These results highlight stCluster’s proficiency in identifying tissue structures within embryos.

**Figure 6:**
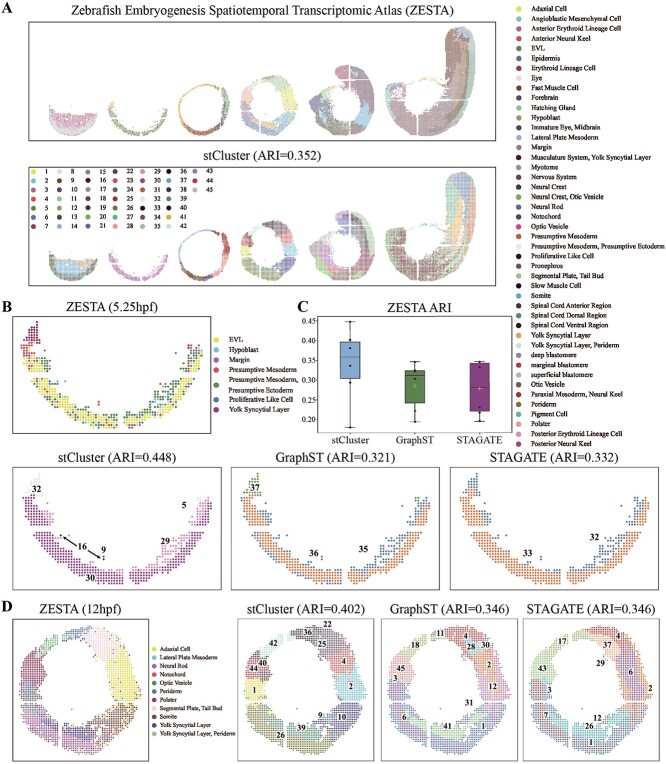
stCluster effectively improves the identification of tissue structures in the zebrafish embryo. **(A)** The manual annotation and the spatial domain detection result visualization. **(B)** The manual annotation and the clustering result of stCluster, GraphST, and STAGATE at the 5.25hpf time point. **(C)** The box plot of the ARI score of stCluster, GraphST, and STAGATE at six-time points. The lower and upper hinges show the first and third quartiles, and the center is the median. Whiskers extend up to 1.5 times the interquartile range from the hinges. Data beyond whiskers are plotted separately. **(D)** The manual annotation and the clustering result of stCluster, GraphST, and STAGATE at the 12hpf time point.

ZESTA is a comprehensive dataset capturing the dynamic changes in gene expression during zebrafish embryogenesis [40]. We employed the complete set of six crucial time points spanning the first 24 hours post-fertilization (hpf), specifically at 3.3hpf, 5.25hpf, 10hpf, 12hpf, 18hpf, and 24hpf, which together comprise 13 166 data spots. Leveraging manual annotations as a reference, we compared the clustering outcomes from stCluster against those derived from GraphST and STAGATE (as depicted in Fig. 6A and Supplementary Figure S3A). Taking all six-time points with 45 clusters for evaluation, stCluster achieves the best performance (ARI = 0.352) compared with GraphST (ARI = 0.311) and STAGATE (ARI = 0.310). We also split the performance at each development time point, as shown in Fig. 6C; stCluster consistently outperforms in most slices (Supplementary Figure S3, S4). Figure 6B shows the embryo at the 5.25hpf time point, consisting of six major cell types. stCluster and GraphST successfully identified the Yolk Syncytial Layer in the upper left region of the embryo, while STAGATE incorrectly merged it with other tissue structures. Furthermore, stCluster distinctly delineates all six domains in alignment with the manual annotation, whereas GraphST and STAGATE manage to recognize only three and two domains, respectively. Observing Fig. 6D, which illustrates the embryo at the 12hpf stage, stCluster once again stands out in its precision, offering clear spatial domains. Notably, stCluster adeptly visualizes the somite domain (as illustrated in stCluster’s domain 10), a region that remains undetected by both GraphST and STAGATE. Furthermore, stCluster precisely identifies the polster structure (represented as domain 26), a structure that gets conflated by the other two methods.

Although stCluster achieves the best performance among the three methods evaluated, the ARI score remains relatively low. We attribute this to the absence of a specific design mechanism for integrating multiple slices, which impedes stCluster and other methods from effectively learning both inner-slice and cross-slice features. To address this limitation, we plan to design a muti-slice joint learning model in our future work.

### stCluster enhances the spatial gene expression patterns

In order to further assess the expressiveness of the latent representations acquired by stCluster, we assessed its performance in gene expression denoising. For this purpose, we developed a denoising model consisting of a single GAT layer and a fully connected network as the decoder. This model utilized the learned representation vectors obtained from stCluster as input and optimized its parameters by minimizing the mean squared error loss between the reconstructed gene expression (obtained from the decoder) and the original gene expression. The resulting gene expression profiles from the decoder were then considered as the denoised gene expression profiles.

**Figure 7:**
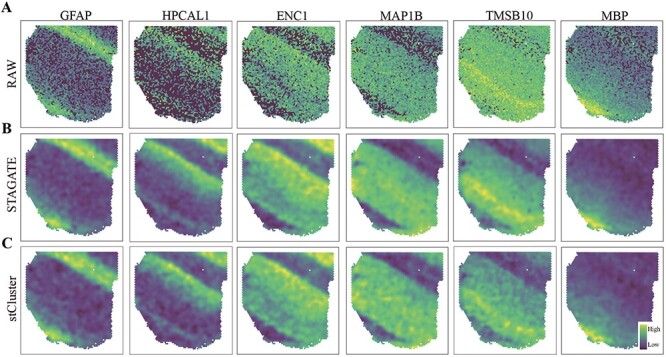
stCluster enhances the spatial gene expression patterns on the DLPFC dataset. The spatial expression patterns of six marker genes based on the raw gene expression **(A)**, STAGATE-denoised gene expression **(B)**, and stCluster-denoised gene expression **(C)**.

In accordance with the experiments conducted in the STAGATE research [20], we proceeded to visualize the spatial expression patterns of six marker genes associated with different layers of the DLPFC (slice 151507), both before and after denoising. These visualizations are presented in Fig. 7. Notably, stCluster demonstrates comparable performance with STAGATE in improving the spatial gene expression patterns. STAGATE is widely recognized for its advanced capability in denoising and imputing spatially resolved transcriptomics data. For instance, after denoising, both methods showcase the differential expression patterns within marker gene HPCAL1 in the layer 2 (Fig. 6B), as opposed to the raw expression patterns (Fig. 7), thereby affirming stCluster’s effectiveness in enhancing spatial gene expression patterns.

### Both contrastive learning and multi-task learning optimizations contribute to the accurate spatial domain identification

**Figure 8:**
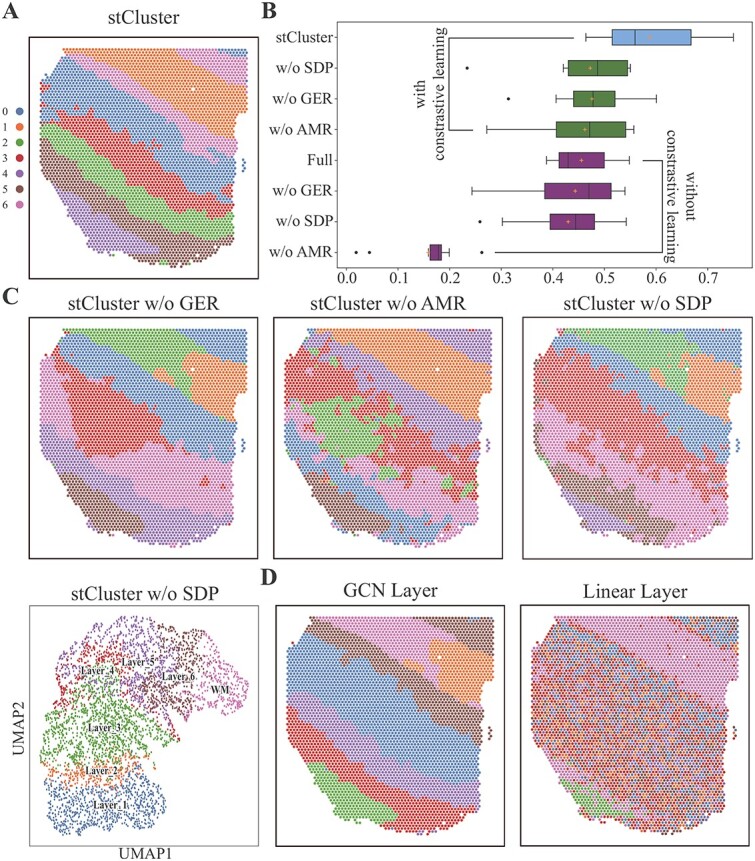
Evaluation of stCluster’s performance after changing the optimization strategies and the network structure. **(A)** The spatial domains identified by stCluster on DLPFC slice 151507. **(B)** The clustering performance of stCluster on DLPFC slice 151507 across different scenarios. The blue boxplot represents stCluster’s complete ability, incorporating all modules. The green boxplots correspond to performance without the SDP, GER, and AMR modules individually. The purple boxplots depict scenarios where the contrastive learning module is excluded. Whiskers extend up to 1.5 times the interquartile range from the hinges, with any data beyond the whiskers plotted separately. **(C)** The spatial domain detection results of stCluster without GER, AMR, and SDP modules individually. The last figure represents the UMAP plot of representations derived by stCluster without the SDP module. **(D)** The spatial domain detection results of stCluster by replacing the GAT layer with the GCN layer and linear layer, respectively.

While the experimental findings highlight the state-of-the-art performance of stCluster in spatial domain identification, it is crucial to quantitatively evaluate the individual contributions of its key components, namely, graph contrastive learning optimization and multi-task learning optimization. Specifically, within the context of the DLPFC dataset sections (Fig. 8A), we observe a significant decline in the average performance of stCluster when the contrastive learning optimization is removed (Fig. 8B). This comparison is assessed against the ”Full” model, which incorporates all three optimization tasks but excludes contrastive learning. It is evident that the contrastive learning module plays a vital role by leveraging vast amounts of unlabeled data to learn meaningful representations. Without this module, stCluster’s performance is compromised.

Indeed, it is important to highlight that the optimizations on all three tasks (GER, SDP, and AMR) are also crucial for stCluster’s performance. This significance is demonstrated by the green boxplots in Fig. 8B, which represent the clustering performance of stCluster after removing each individual optimization task. It is evident from the results that removing any one of the optimization tasks leads to a significant decline in stCluster’s performance. Therefore, both contrastive learning and multi-task optimization play an integral role in ensuring the effectiveness of stCluster in spatial domain identification.

Furthermore, we assessed the impact on clustering when each individual optimization task was removed, as depicted in Fig. 8C. Removing the GER task causes the single layer to separate into distinct clusters. The absence of the AMR task results in vague boundary delineation. Likewise, the absence of the SDP task contributes to both phenomena and disrupts the clear spatial arrangement of spots, as evident in the UMAP visualization (compared with Figure 2D). We also experimented with replacing the GAT layer in our model with a GCN layer and a linear fully connected layer. As expected, we observe a decline in the clustering effect, as depicted in Fig. 8D. It is evident from these observations that both the optimization strategies and network structures play critical roles in the task of spatial domain identification.

## Conclusions

ST sequencing has become a powerful tool that allows mapping and analysis of gene expression patterns within the spatial context. In ST, a crucial task is spatial domain identification, where the objective is to cluster spots or cells with similar spatial expression patterns into biologically meaningful spatial domains. However, existing methods encounter difficulties in effectively integrating gene expression data with spatial information, leading to less informative representations and suboptimal accuracy.

To address these challenges, we propose a novel graph deep learning model called stCluster. Our model is specifically designed to learn enhanced representations of spots by effectively integrating gene expression with spatial information, ultimately improving the accuracy of spatial domain identification. To achieve this purpose, stCluster incorporates two key strategies, namely graph contrastive learning and multi-task learning, in its model optimization. By leveraging graph contrastive learning, stCluster encourages the model to capture the underlying meaningful representations and distinguish between different spatial expression profiles by utilizing abundant unlabeled spots. In addition to contrastive learning, stCluster employs multi-task learning to further enhance its performance, including the tasks of GER, SDP, and AMR. By jointly optimizing these tasks, stCluster improves its ability to capture the complex relationships between gene expression and spatial organization.

Experimental findings indicate that stCluster demonstrates better performance compared with six state-of-the-art methods across various datasets and sequencing platforms. Employing the 12 DLPFC slices as a benchmark, stCluster exhibits an average accuracy enhancement of 16% over the next best-performing method. It exhibits better accuracy in identifying tissue spatial structures across various levels, including tissue-level slices (such as the human prefrontal cortex, mouse olfactory bulb, and mouse somatosensory cortex), organ-level slices (such as the mouse brain), and embryo-level slices (such as the zebrafish embryo and mouse embryo). Moreover, stCluster also excels in denoising and enhancing spatial gene expression patterns. Furthermore, we illustrated the pivotal role of the combined optimization strategies and neural network structures in stCluster’s performance. Additionally, we have assessed the computational resource consumption of stCluster compared with other state-of-the-art methods. This evaluation encompassed the analysis of RAM usage, time consumption, and GPU memory consumption, with detailed results presented in Supplementary Figure S6. The findings indicate that stCluster effectively balances performance with computational resource efficiency, demonstrating its practical applicability in diverse settings.

However, stCluster encounters two primary challenges. Firstly, it grapples with the issue of sensitivity to initial parameters, a common concern in deep learning frameworks [46]. The random initialization of parameters may influence convergence stability, despite attempts to mitigate this by setting random seeds in our framework. Secondly, current ST clustering methods, including stCluster, face difficulties in effectively handling multi-slice data. To address this limitation, we intend to develop a new multi-slice integration framework in our future work.

In conclusion, stCluster emerges as one of state-of-the-art techniques in the realm of representation learning for ST data, providing an innovative approach to identify and delineate spatial domains. Moreover, as the field of ST continues to evolve, we envision stCluster becoming a useful tool for researchers working in this domain.

**Key Points**

- This work presents stCluster, which can learn informative representations and accurately identify spatial domains for spatially resolved transcriptomics.
- stCluster collaboratively utilizes graph contrastive learning and multi-task learning techniques to effectively integrate gene expression and spatial information reserved in spatial transcriptomic data.
- Experimental results demonstrate that stCluster improves the clustering performance compared with existing state-of-the-art techniques.
- stCluster is robust to various scales of spatial transcriptomic data, spanning tissue, organ, and embryo levels and consistently performs well across sequencing platforms.
- stCluster is also able to enhance the spatial gene expression patterns and the spatial trajectory inferences.

## Supplementary Material

Supplementary_Information_bbae329

## Data Availability

All data used in this article can be obtained at our GitHub repository: https://github.com/hannshu/st_datasets.

## Author Biographies

**Tao Wang** is an associate professor in Bioinformatics and Artificial Intelligence at the Northwestern Polytechnical University. His research interests include the development and application of bioinformatics methods for the analysis of genomics data.

**Han Shu** is a graduate student in the School of computer science at the Northwestern Polytechnical University. His research activity focuses on the development of computational methods for analyzing transcriptomics data.

**Jialu Hu** is an associate professor in Computer Science at the Northwestern Polytechnical University. His research activities are mainly focused on data analysis and developing deep-learning methods in the biological field.

**Yongtian Wang** is an associate professor in Bioinformatics at the Northwestern Polytechnical University. His research activities are mainly focused on the development and application of methods for Bioinformatics.

**Jing Chen** is a lecturer in Computer Science at the Xi’an University of Technology. Her research interests mainly focus on artificial intelligence methods in bioinformatics and brain sciences.

**Jiajie Peng** is an associate professor in Bioinformatics and Artificial Intelligence at the Northwestern Polytechnical University. His research interests include the development and application of bioinformatics methods for the analysis of genomics and single-cell sequencing data.

**Xuequn Shang** is a professor in Bioinformatics and Big Data Analysis at the Northwestern Polytechnical University. Her research activities are mainly focused on the development and application of methods for biomedical, educational, and financial big data.
