# Supplementary material for: Accurately deciphering spatial domains for spatially resolved transcriptomics with stCluster
Source: Brief Bioinform. 2024 Jul 8;25(4):bbae329. doi: 10.1093/bib/bbae329 (PMC11771244; doi:10.1093/bib/bbae329)
Supplement: Supplementary_Information_bbae329 [file supplementary_information_bbae329.pdf]

Supplementary Information

Accurately deciphering spatial domains for spatially resolved transcriptomics with stCluster

Tao Wang[1,2,†,*], Han Shu[1,2,†], Jialu Hu[1,2], Yongtian Wang[1,2], Jing Chen[3,*], Jiajie Peng[1,2,*], and Xuequn Shang[1,2,*]

[1]School of Computer Science, Northwestern Polytechnical University, Xi'an, 710072, China

[2]Key Laboratory of Big Data Storage and Management, Northwestern Polytechnical University, Ministry of Industry and Information Technology, Xi'an, 710072, China

[3]School of Computer Science and Engineering, Xi'an University of Technology, Xi'an, 710048, China

[†]These authors contributed equally to this work

[*]To whom correspondence should be addressed. Emails: T.W. twang@nwpu.edu.cn; X.S. Shang@nwpu.edu.cn; J.C. chen-jing@xaut.edu.cn; J.P. jiajiepeng@nwpu.edu.cn

CONTENTS

Supplementary Note 1 Hyperparameter Evaluation 4

Figure S1-1 Hyperparameters tuning test result in the mouse brain serial section 1 anterior dataset. 4

Figure S1-2 The clustering result for stCluster by different clustering algorithms in the mouse brain serial section 1 anterior dataset. 5

Figure S1-3 Utilize clustering methods on two unlabeled human breast cancer datasets and compare the clustering results obtained using Mclust and Louvain algorithms, respectively. 6

Figure S1-4 The clustering result for stCluster by different input HVGs in the mouse brain serial section 1 anterior dataset. 6

Supplementary Note 2 Comparison of the Model Structures Between stCluster and State-of-the-art Methods 7

Supplementary Note 3 Measuring clustering result using ARI, NMI, and IoU 9

Figure S1 Manual annotations and spatial domain identifications of SEDR, SpaGCN, STAGATE, CCST, DeepST, GraphST, and stCluster on the 12 slices of DLPFC dataset, respectively. 11

Figure S2 stCluster accurately distinguishes different structures in the mouse brain. 12

Figure S3 Manual annotations and spatial domain identifications of STAGATE, GraphST, and stCluster on the Zebrafish Embryogenesis Spatiotemporal Transcriptomic Atlas and 3.3 hpf data. 13

Figure S4 Manual annotations and spatial domain identifications of STAGATE, GraphST, and stCluster on the Zebrafish Embryogenesis 10 hpf, 16 hpf, and 24 hpf data, respectively. 14

Figure S5 Manual annotations and spatial domain identifications of STAGATE, GraphST, and stCluster on the Mouse Organogenesis Spatiotemporal Transcriptomic Atlas E9.5 data, respectively. 15

Figure S6 The time assumption, RAM cost, and GPU memory cost of all methods

in DLPFC dataset, respectively. 16

Table S1. The state-of-the-art methods for embedding learning and spatial domain identification in spatial transcriptomics. 17

Table S2. The model structure comparison of stCluster and the state-of-the-art methods. 18

Table S3. Summary of all spatial transcriptomic datasets used for experiments in this work. # means the number of. 20

Table S4. The summary of clustering performance (ARI scores) on 12 slices of the DLPFC dataset derived by seven methods. 22

Table S5. The summary of clustering performance (NMI scores) on 12 slices of the DLPFC dataset derived by seven methods. 22

Table S6 The summary of clustering performance based on the Intersection over Union (IoU) metric on 12 slices of the DLPFC dataset derived by seven methods. 23

Reference 24

Supplementary Note 1 Hyperparameter Evaluation

We evaluate the hyperparameter used in stCluster. The hyperparameters used in stCluster are set based on prior knowledge, empirical observations, or theoretical considerations. In this section, we mainly evaluate the number of neighbors in SAG (Fig. S1-1 A), the preclusted graph neighbors k (Fig. S1-1 B), the preclusted graph drop rate θ (Fig. S1-1 C), the contrastive learning cutting probability c_k (Fig. S1-1 D), and the multi-task optimization loss weight α_{adj}, α_{gene}, and α_{pred} (Fig. S1-1 E) in the mouse brain serial section 1 anterior dataset.

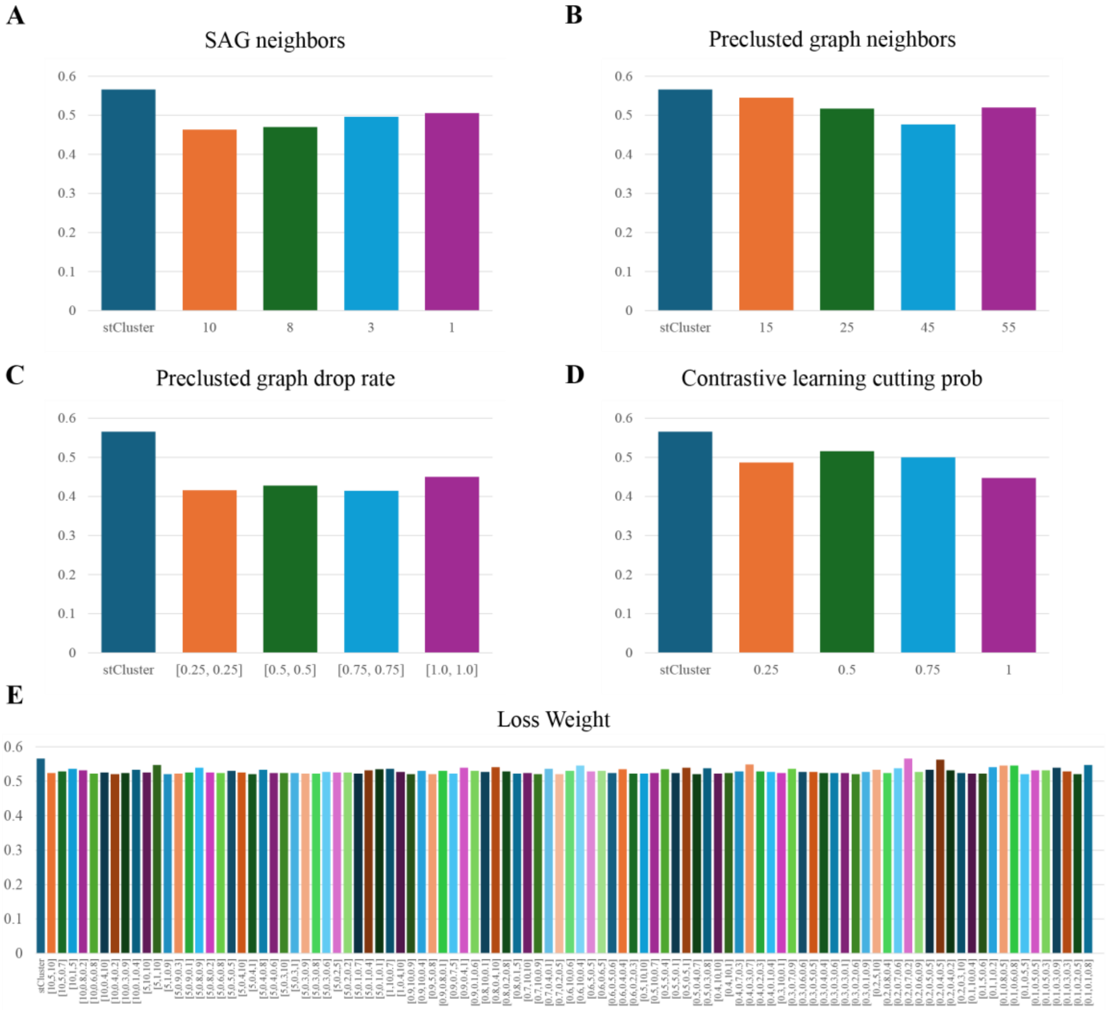

Figure S1-1 Hyperparameters tuning test result in the mouse brain serial section 1 anterior dataset. (A) The clustering result for stCluster by different numbers of neighbors in SAG in the mouse brain serial dataset. (B) The hyperparameters tuning result of the preclusted graph neighbors k. (C) The hyperparameters tuning result of the preclusted graph drop rate θ. (D) The hyperparameters tuning result of the contrastive learning cutting probability c_k. (E) The hyperparameters tuning results of the loss weight in multi-task optimization process, the x axes are the α_{adj}, α_{gene}, and α_{pred}, respectively.

For the number of neighbors in SAG, k, θ, and c_k, we have selected four parameters for each and observed the impact of each parameter on the model performance while keeping the other parameters constant. For the multi-task optimization loss weight (α_{adj}, α_{gene}, and α_{pred}), we have selected 50 possible parameter combinations and observed the running performance of the model while keeping the other parameters constant. From the results, we can see that even the selection of hyperparameters will affect the model performance (which is also the case for most deep-learning models), the default parameters of stCluster can achieve a better performance, and the change of hyperparameters will not dramatically affect the performance.

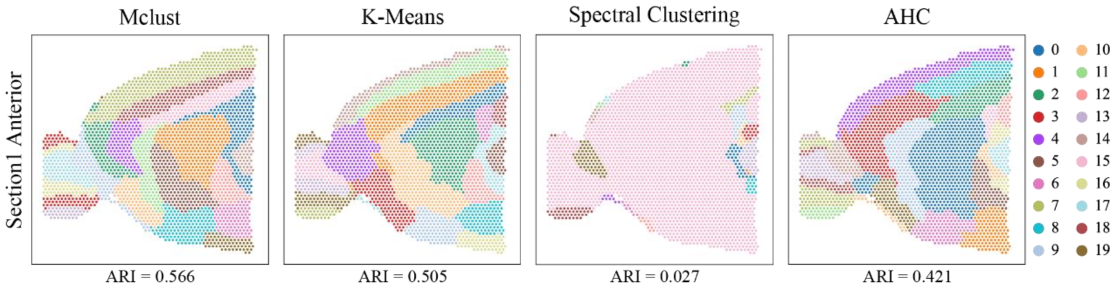

Figure S1-2 The clustering result for stCluster by different clustering algorithms in the mouse brain serial section 1 anterior dataset. The Mclust, K-Means, Spectral clustering, and Agglomerative Hierarchical Clustering (AHC) algorithms for representation of the mouse brain serial dataset, respectively.

Furthermore, we assess the clustering performance of various clustering algorithms. We utilize the representation learned by stCluster and employ different methods, including mclust (a Gaussian mixture model-based clustering method) [1], K-Means (a centroid-based clustering method) [2], Spectral clustering [3], and AHC (a hierarchical clustering method) [4] to evaluate the results (Fig. S1-2). We find that the Mclust algorithm can achieve the greatest result.

Moreover, when dealing with unlabeled datasets, we also evaluate the clustering performance of the Louvain [5] community partition algorithm, which does not require setting the number of clusters (Fig. S1-3). The result shows that stCluster can perform better among the three methods using Mclust or Louvain clustering algorithms. This is evaluated by the Silhouette coefficient [6] (SC), which assesses the quality of a clustering result. SC evaluates how similar an object is to its cluster compared to other clusters, and the SC score ranges from -1 to 1, where a higher score indicates that the sample is well matched to its cluster.

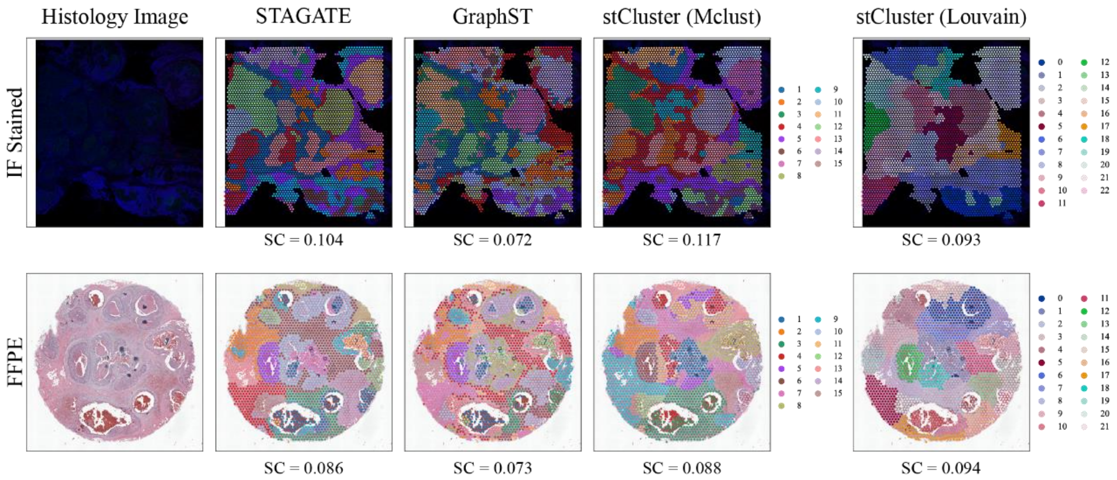

Figure S1-3 Utilize clustering methods on two unlabeled human breast cancer datasets and compare the clustering results obtained using Mclust and Louvain algorithms evaluated by the Silhouette coefficient, respectively.

Additionally, we also assess the impact of the input size on our model in the mouse brain serial section1 anterior dataset (Fig. S1-4).

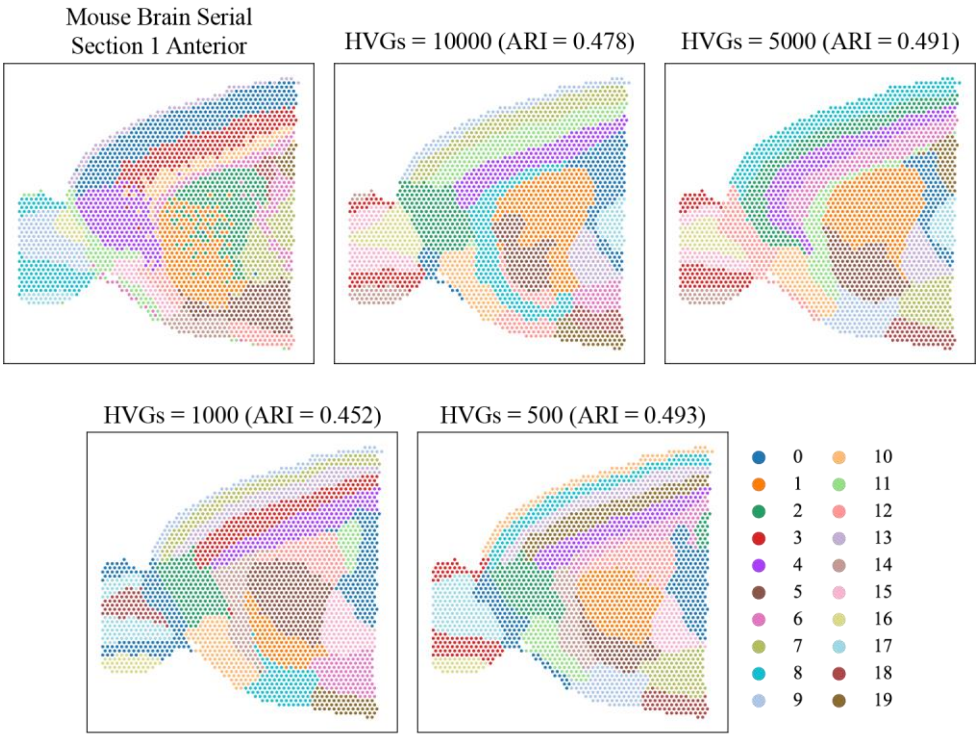

Figure S1-4 The clustering result for stCluster by different input HVGs in the mouse brain serial section 1 anterior dataset.

Supplementary Note 2 Comparison of the Model Structures Between stCluster and State-of-the-art Methods

To inspect the similarities and differences between stCluster and other state-of-the-art methods for spatial domain identification, we separate the computational process into six main steps and compare them step by step. The main steps include constructing cell neighborhood graphs, encoders, decoders, optimization strategies, contrastive learning strategies (if applicable), clustering methods, and have been summarized in Supplementary Table S2.

In the initial step of constructing the spot neighborhood graph, all methods employ K-nearest neighbors algorithms[7] to generate the spatial graph used in the graph neural network. SpaGCN[8] utilizes a unique algorithm designed specifically for graph construction. GraphST[9] sets the number of nearest neighbors to four, whereas SEDR [24] is normally set to 12 . Conversely, CCST[10] restricts users to constructing spatial graphs using only a preset radius (in the DLPFC dataset, we set it to 150). Meanwhile, STAGATE[11], DeepST[12], and stCluster provide users with the flexibility to construct spatial graphs based on either the K nearest neighbors or a specified radius.

Our method and several compared methods employ auto-encoder[13] structures to learn representations for each spot. Both SEDR and DeepST utilize Variational Autoencoders (VAE)[14], incorporating GCN layers[15] and fully connected layers to construct the encoder, and using fully connected layers for the decoder. STAGATE employs both GAT layers[16] and fully connected layers in its encoder and decoder. GraphST uses GCN layers in both the encoder and decoder, while stCluster employs GAT layers and fully connected layers in the encoder and only fully connected layers in the decoder. Notably, SpaGCN and CCST diverge from this trend; SpaGCN leverages only the GCN layer for representation learning, and CCST uses the DGI model[17], with neither method employing the autoencoder technique.

During the model optimization step, various strategies are employed to optimize the model parameters for enhanced representation learning. SEDR and DeepST utilize a combination of MSE (Mean Squared Error) loss[18], Binary Cross Entropy loss[19], and KL-divergence loss[20]. SpaGCN specifically employs KL-divergence loss for parameter optimization. In contrast, STAGATE uses MSE loss to optimize the congruence between the outputs of the autoencoder and the input gene expression data. CCST, on the other hand, optimizes using the loss function specific to the DGI model. GraphST employs both MSE loss and Noise Cross Entropy loss for its optimization processes. Lastly, stCluster utilizes Contrastive Learning loss alongside Multi-task Optimization loss to fine-tune its model parameters.

CCST, GraphST, and stCluster incorporate contrastive learning strategies to optimize their model parameters. Both CCST and GraphST utilize the DGI contrastive learning strategy, which aims to maximize the similarity between representations in positive and negative graphs. On the other hand, stCluster employs a GCA-based contrastive learning strategy, focusing on maximizing self-similarity while minimizing both internal similarity and cross-view similarity.

In the clustering step, methods utilize their learned representations as input to a clustering algorithm to generate the final clustering results. Specifically: SEDR employs the Leiden algorithm[21] to obtain its clustering outcomes. SpaGCN applies the DEC (Deep Embedded Clustering) algorithm[22] as integrated within its loss function to produce clustering results. CCST leverages the K-Means algorithm[2] for clustering. DeepST uses the Louvain algorithm[5]

to derive its clustering results. Both STAGATE, GraphST, and stCluster utilize the Mclust-EEE[1] function to generate their clustering outcomes.

Supplementary Note 3 Measuring clustering result using ARI, NMI, and IoU

Adjusted Rand Index

The Adjusted Rand Index (ARI)[23] is a measure used to evaluate the similarity between two partitions, or, in other words, two set clustering results, of the same dataset, adjusting for the chance grouping of elements. It is particularly useful in the context of assessing the performance of clustering algorithms.

Give a set of n elements with two partitions $X = \{X_1, X_2, \ldots, X_r\}$ and $Y = \{Y_1, Y_2, \ldots, Y_r\}$. The overlap between each partition X_i and Y_j denote as n_{ij}, where $n_{ij} = |X_i \cap Y_j|$. The ARI score can be calculated as follows:

$$ARI = \frac{\sum_{ij}\binom{n_{ij}}{2} - [\sum_i \binom{a_i}{2} \sum_j \binom{b_j}{2}]/\binom{n}{2}}{\frac{1}{2}[\sum_i \binom{a_i}{2} + \sum_j \binom{b_j}{2}] - [\sum_i \binom{a_i}{2} \sum_j \binom{b_j}{2}]/\binom{n}{2}} \quad (1)$$

Where $a_i = \sum_j n_{ij}$ for each partition X_i and $b_j = \sum_i n_{ij}$ for each partition Y_j.

While the original Rand Index[24] ranges from 0 to 1, where 1 indicates perfect agreement between two partitions, the ARI can also take negative values. This occurs when the observed agreement is less than what would be expected by chance, indicating a clustering agreement worse than random.

Normalized Mutual Information

The Normalized Mutual Information (NMI)[25] is a measure used to evaluate the mutual information between two sets. The NMI score can be calculated as follows:

$$NMI = \frac{2 \times I(Y; C)}{[H(Y) + H(C)]} \quad (2)$$

Where Y represents the ground truth labels and C represents the predicted cluster results. The function $H(\cdot)$ measures the entropy of the input labels, while $I(Y;\ C)$ represents the mutual information between the ground truth labels and the clustering results.

The scale of the NMI score ranges from 0 to 1, where 0 indicates that the two sets Y and C have no mutual information, and 1 indicates a complete match between the two sets.

Clustering Intersection over Union

We also evaluated the clustering performance based on the Intersection over Union (IoU)[26] metric tailored to the spatial domain clustering scenario based on the human DLPFC dataset. We began by calculating the Intersection over Union (IoU) score for each ground truth label A_i and the corresponding cluster region B_i as follows:

$$IoU_i = \frac{|A_i \cap B_i|}{|A_i \cup B_i|} \quad (3)$$

This helped us construct a relationship matrix, where each row represented a clustering result,

and each column represented a ground truth label as follows. Next, we used the Hungarian algorithm[27] to find the optimal assignment between the clustering results and the labels. By maximizing the mean of IoU scores for each column, we determined the best matching between the two sets. The final IoU score is calculated as the average IoU across all clusters.

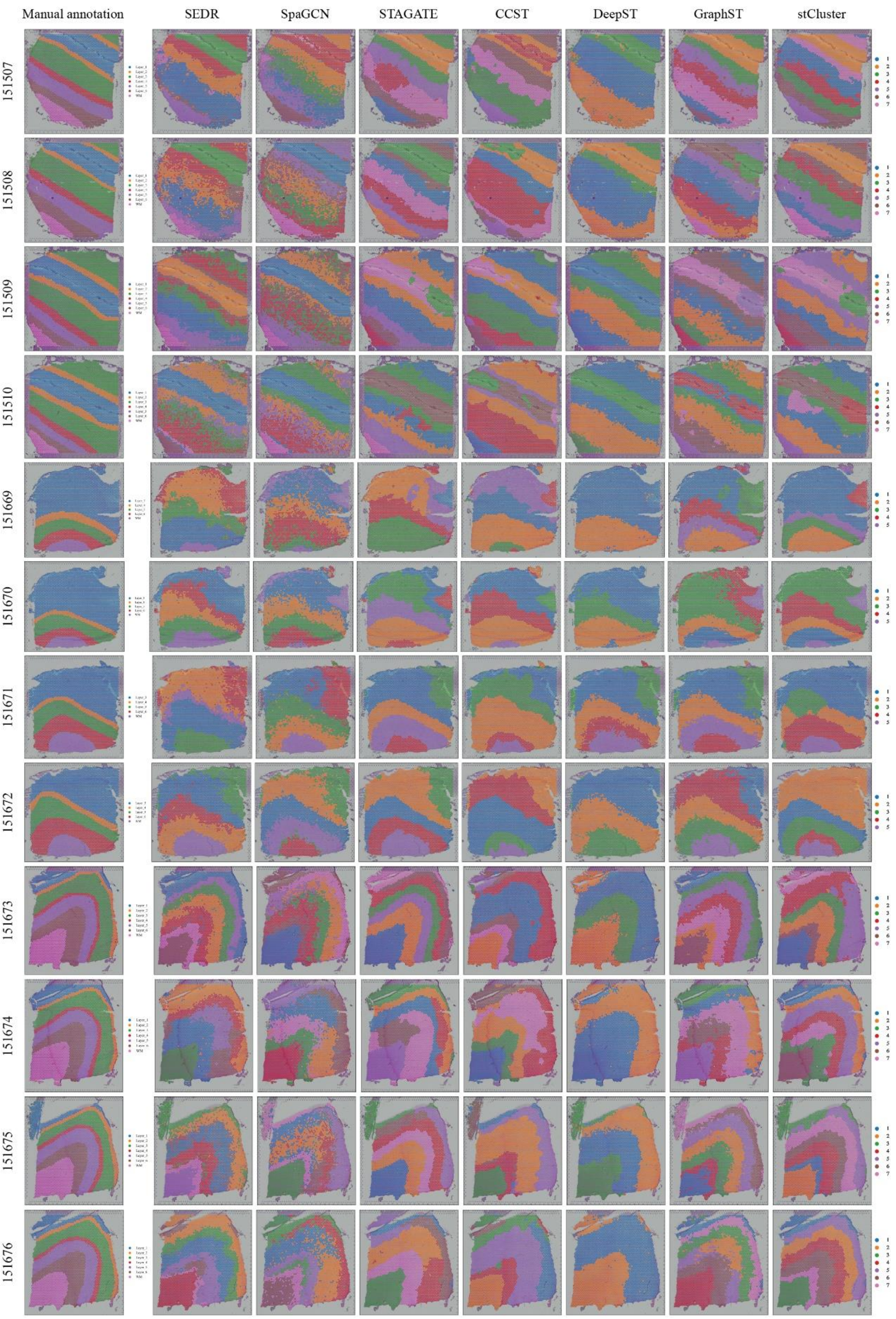

Figure S1 Manual annotations and spatial domain identifications of SEDR, SpaGCN, STAGATE, CCST, DeepST, GraphST, and stCluster on the 12 slices of DLPFC dataset, respectively.

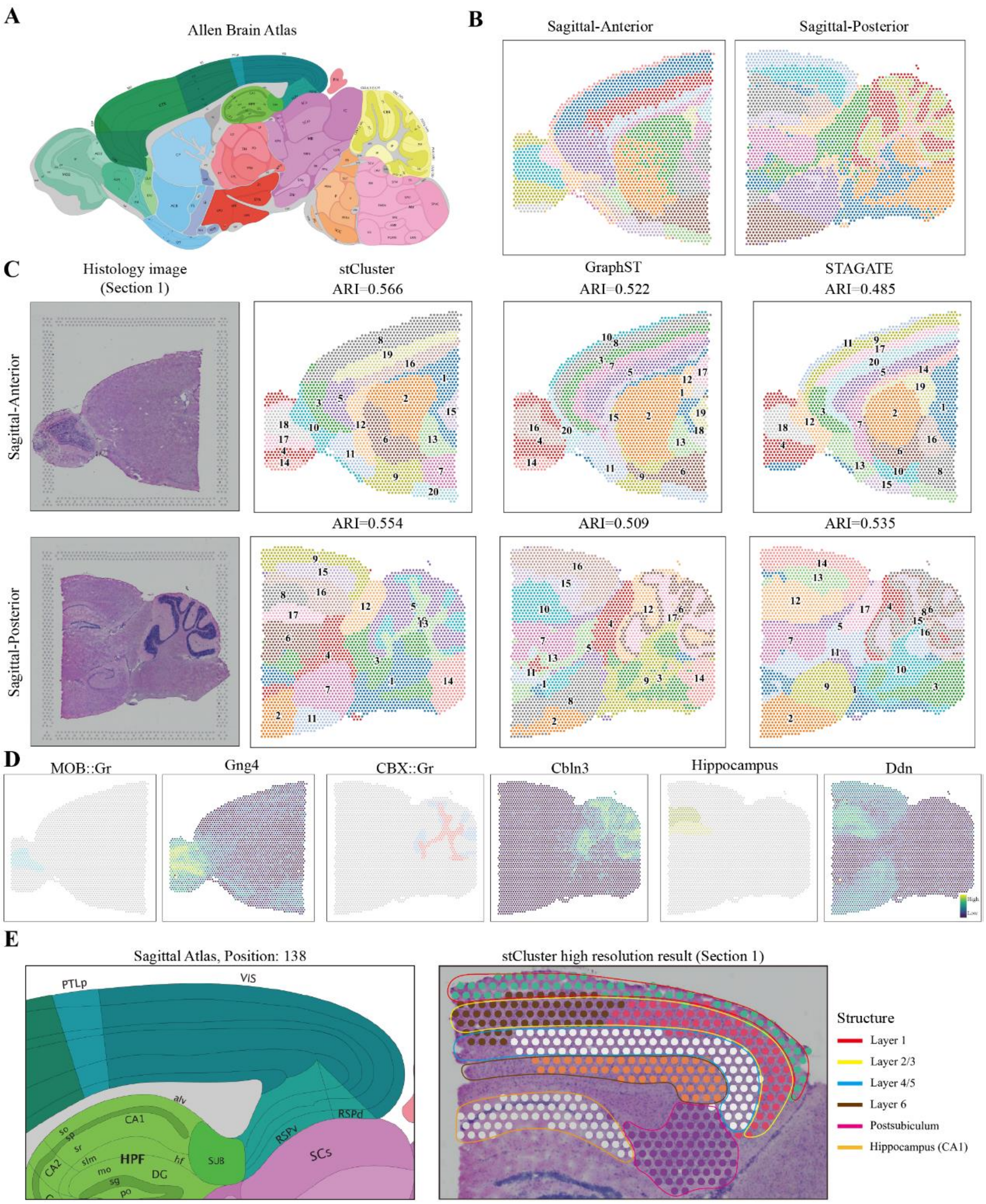

Figure S2 stCluster accurately distinguishes different structures in the mouse brain. (A) The Allen brain atlas of the sagittal mouse brain in position 138. (B) The manual annotation of mouse brain serial dataset section 1 (Anterior and Posterior). (C) Row 1: The histology image, spatial domain identification of stCluster, GraphST, and STAGATE in section 1 anterior slice, respectively; Row 2: The histology image, spatial domain identification of stCluster, GraphST, and STAGATE in section 1 posterior slice, respectively. (D) The spatial distribution of structures MOB::Gr, Hippocampus, and CBX::Gr stCluster predicted and the corresponding marker genes Gng4, Cbln3, and Ddn, respectively. (E) Visual areas and hippocampal formation of the Allen brain atlas in position 138 (left), the high-resolution clustering result, and the manual annotation to identify finer-grained structures by stCluster in mouse brain serial sagittal-posterior section 2 slices (right).

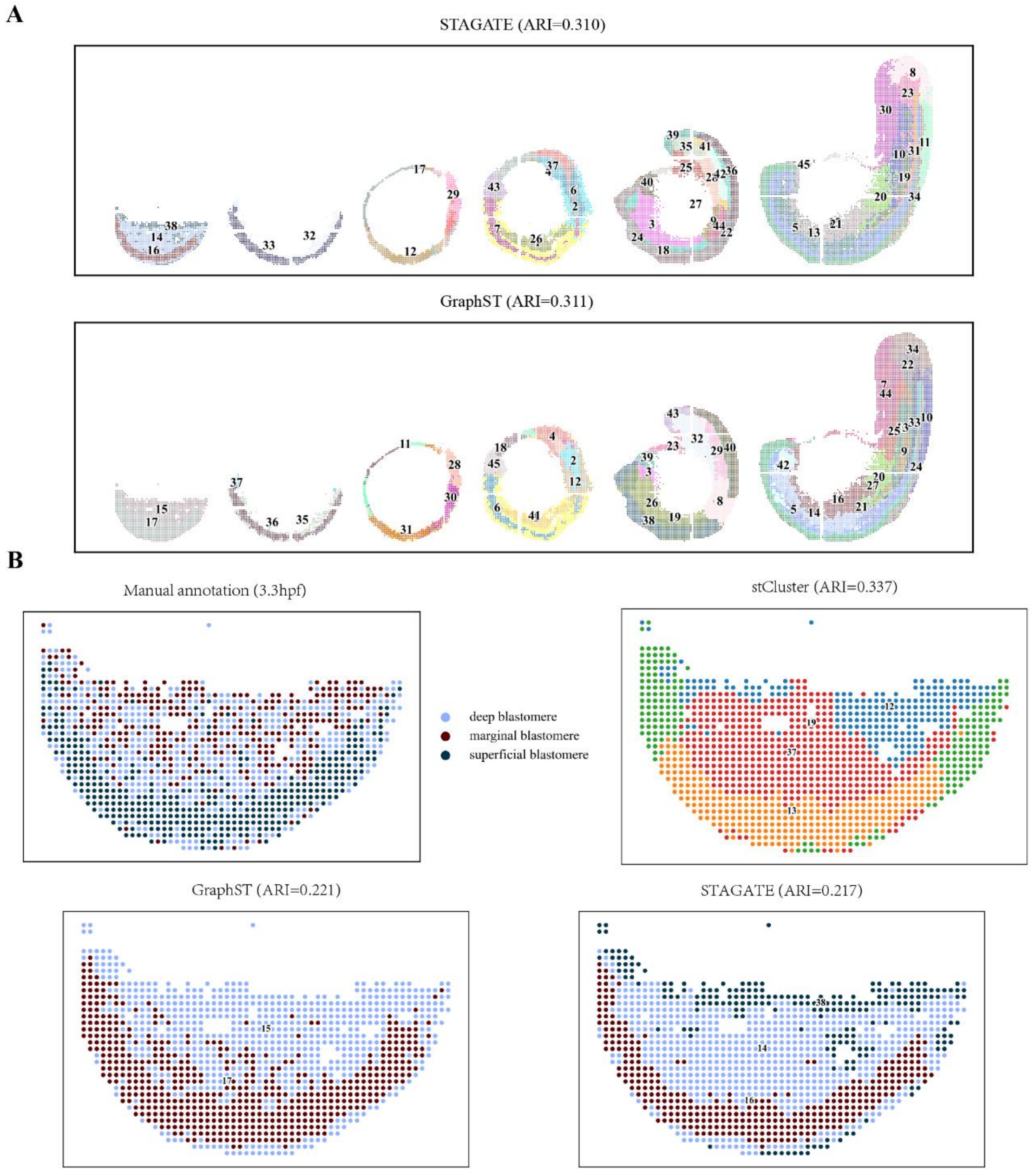

Figure S3 Manual annotations and spatial domain identifications of STAGATE, GraphST, and stCluster on the Zebrafish Embryogenesis Spatiotemporal Transcriptomic Atlas and 3.3 hpf data. (A) domain identification results of STAGATE and GraphST on six-time zebrafish embryo dataset. (B) domain identification results of STAGATE, GraphST, and stCluster on 3.3 hpf zebrafish embryo dataset.

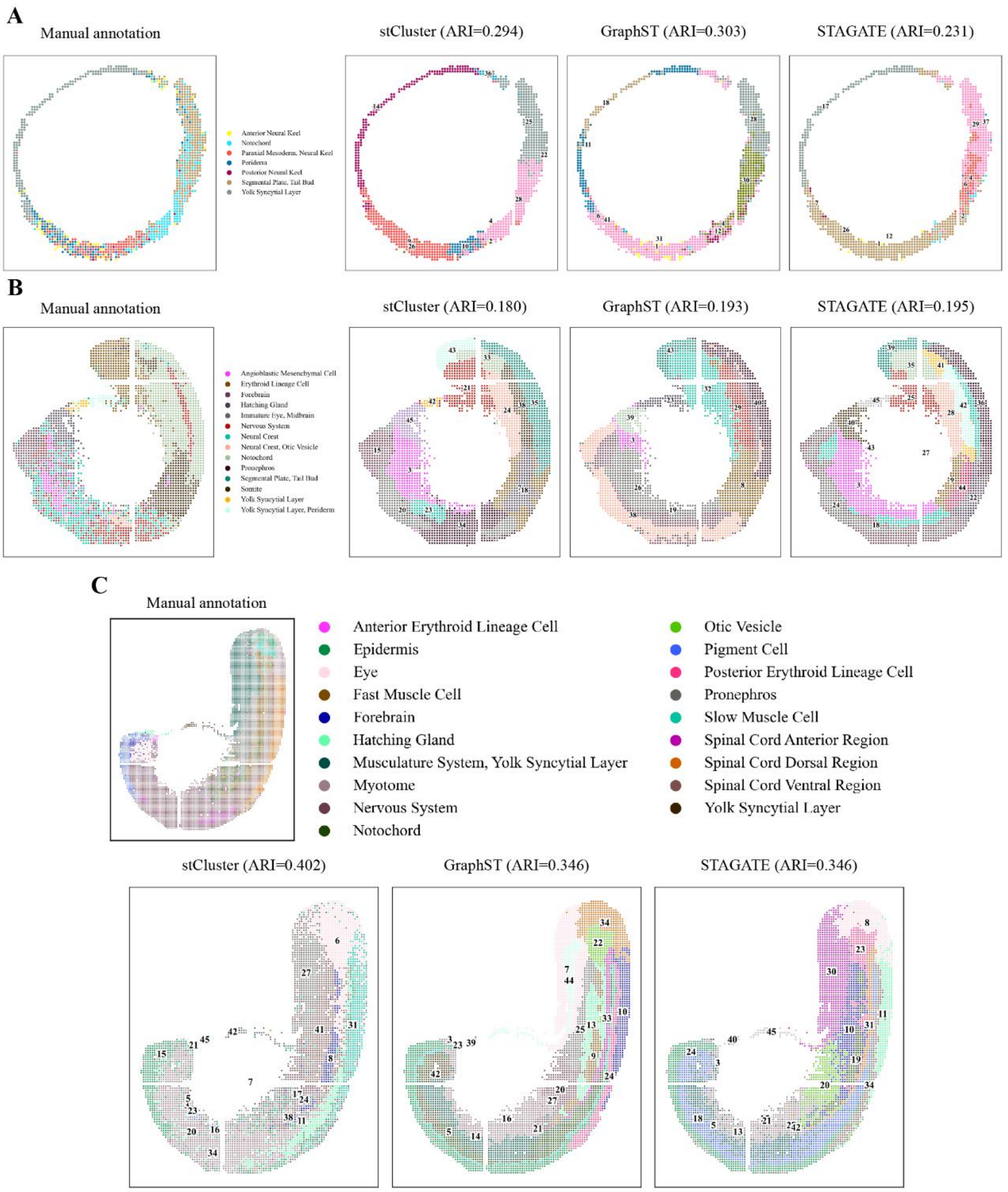

Figure S4 Manual annotations and spatial domain identifications of STAGATE, GraphST, and stCluster on the Zebrafish Embryogenesis 10 hpf, 16 hpf, and 24 hpf data, respectively. (A) domain identification results of STAGATE, GraphST, and stCluster on 10 hpf zebrafish embryo dataset. (B) domain identification results of STAGATE, GraphST, and stCluster on 16 hpf zebrafish embryo dataset. (C) domain identification results of STAGATE, GraphST, and stCluster on 24 hpf zebrafish embryo dataset.

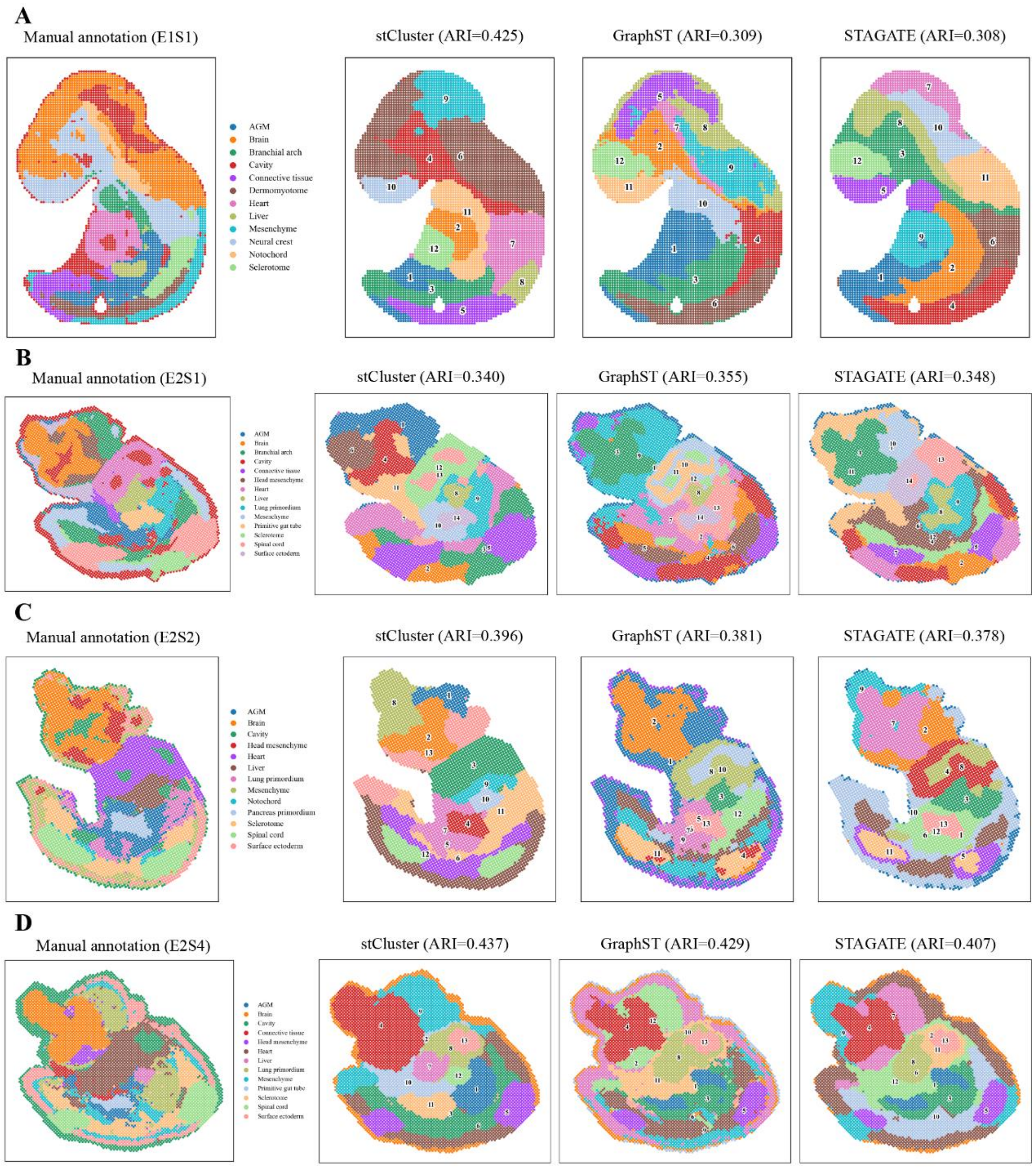

Figure S5 Manual annotations and spatial domain identifications of STAGATE, GraphST, and stCluster on the Mouse Organogenesis Spatiotemporal Transcriptomic Atlas E9.5 data, respectively. (A) domain identification results of STAGATE, GraphST, and stCluster on E1S1 mouse embryo dataset. (B) domain identification results of STAGATE, GraphST, and stCluster on E2S1 mouse embryo dataset. (C) domain identification results of STAGATE, GraphST, and stCluster on E2S2 mouse embryo dataset. (D) domain identification results of STAGATE, GraphST, and stCluster on E2S3 mouse embryo dataset.

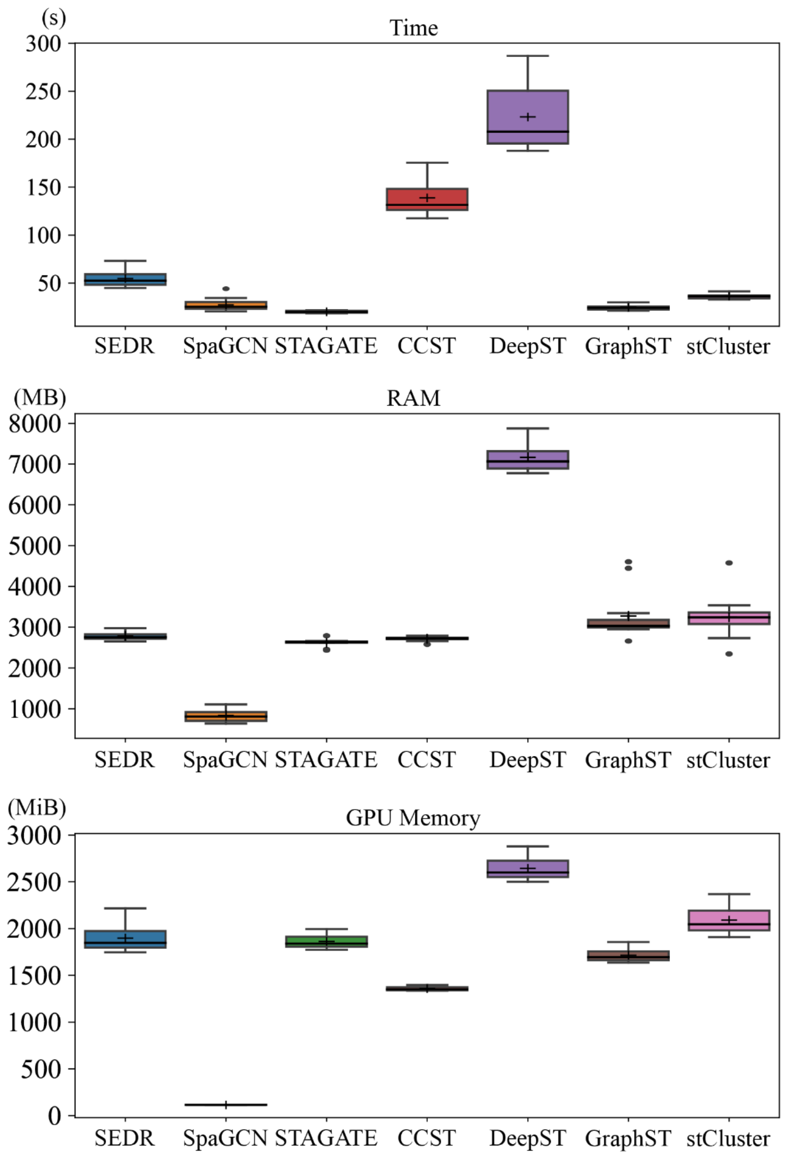

Figure S6 The time assumption, RAM cost, and GPU memory cost of all methods in DLPFC dataset, respectively. The results indicate that stCluster operates with relatively high efficiency, exhibiting fast processing speeds and lower consumption of RAM and GPU memory compared to other methods.

Table S1. The state-of-the-art methods for embedding learning and spatial domain identification in spatial transcriptomics.

Method	Basic model	Use histology image	Source code link	Reference
SEDR	GCN[15], VAE[14]	No	https://github.com/JinmiaoChenLab/SEDR	Chen *et. al.*, bioRxiv, 2021[28]
SpaGCN	GCN	Yes	https://github.com/jianhuupenn/SpaGCN	Hu *et al.*, *Nat. Methods*, 2021[8]
STAGATE	GAT[16], AE[13]	No	https://github.com/QIFEIDKN/STAGATE_pyG	Dong and Zhang, *Nat. Commun.*, 2022[11]
CCST	DGI[17]	No	https://github.com/xiaoyeye/CCST	Li *et al.*, *Nat. Comput. Sci.*, 2022[10]
DeepST	GCN, VAE	Yes	https://github.com/JiangBioLab/DeepST	Xu *et al.*, *Nucleic Acids Res.*, 2022[12]
GraphST	GCN	No	https://github.com/JinmiaoChenLab/GraphST	Long *et al.*, *Nat. Commun.*, 2023[9]

Table S2. The model structure comparison of stCluster and the state-of-the-art methods.

Method	Constructing cell neighborhood graph step		Encoder-utilizing steps	Decoder-utilizing steps	Optimization strategy	Contrastive learning strategy	Clustering steps
	build by k-nearest neighbor	build by radius					
SEDR	Allow	Not Allow	Fully Connected layer and GCN layer	Fully Connected layer	MSE loss[18], Binary Cross Entropy loss[19], and KL-divergence loss[20]	Not applied	Leiden[21]
SpaGCN	Not Allow	Not Allow	GCN layer	Not applied	KL-divergence loss	Not applied	DEC[22]
STAGATE	Allow	Allow	GAT layer and Fully Connected layer	GAT layer and Fully Connected layer	MSE loss	Not applied	Mclust-EEE [1]
CCST	Not Allow	Allow	DGI[17]	Not applied	DGI	DGI	K-Means[2]
DeepST	Allow	Allow	Fully Connected layer and GCN layer	Fully Connected layer	MSE loss, Binary Cross Entropy loss, and KL-divergence loss	Not applied	Louvain[5]
GraphST	Allow	Not Allow	GCN layer	GCN layer	MSE loss and Noise Cross Entropy loss	DGI	Mclust-EEE
stCluster	Allow	Allow	GAT layer and Fully Connected layer	Fully Connected layer	Contrastive Learning loss and Multi-task Optimization loss	maximizing the self-similarity while minimizing internal similarity and cross-view similarity	Mclust-EEE

In our study, we implemented a rigorous selection process to compare different methods, ensuring that our analysis reflects the latest advancements in the field of spatial transcriptomics representation learning and domain detection. We specifically focused on evaluating the most innovative methods published between 2021 and 2023 (our method is submitted in October 2023). While we acknowledge the contributions of similar methodologies like Seurat[29], Giotto[30], stLearn[31], BayesSpace[32], and SpaceFlow[33], we exclude direct comparisons with these methods. This decision was based on the fact that the methods we compared with had already conducted comprehensive evaluations that demonstrated the underperformance of earlier published methods relative to more recent developments. To maintain the scientific rigor and relevance of our analysis, we carefully selected six of the most advanced methods currently available. This selective approach allows us to provide a detailed and contemporary evaluation of state-of-the-art methods, ensuring that our comparisons are both pertinent and valuable to ongoing research in this field.

Table S3. Summary of all spatial transcriptomic datasets used for experiments in this work. # means the number of.

Source	Section ID	#Spots	#Genes	#Clusters	Sequencing technology	Related figures
Human dorsolateral prefrontal cortex (DLPFC)[34]	151507	4226	33538	7	10x Visium	Fig. 2, Fig 7, Fig. 8, Fig. S1, Table S1
	151508	4384		7		
	151509	4789		7		
	151510	4634		7		
	151669	3661		5		
	151670	3498		5		
	151671	4110		5		
	151672	4015		5		
	151673	3639		7		
	151674	3673		7		
	151675	3592		7		
	151676	3460		7		
Mouse olfactory bulb	-	10000	26145	8	Stereo-seq	Fig. 3A
	-	918	31053	8	10x Visium	Fig. 3B
	-	264	15941	4	Spatial Transcriptomics (ST)	Fig. 3C
Mouse somatosensory cortex[35]	-	5328	33	6	osmFISH	Fig. 3D
Mouse brain serial	Section1 Anterior	2696	31053	20	10x Visium	Fig. S2
	Section1 Posterior	3353		17		
	Section2 Anterior	2825				Fig. 4
	Section2 Posterior	3293				
Zebrafish Embryogenesis Spatiotemporal Transcriptomic Atlas[36]	3.3hpf	1167	26628	3	Stereo-seq	Fig. S3B
	5.25hpf	563		7		Fig. 5B
	10hpf	1036		7		Fig. S4A
	12hpf	2081		11		Fig. 5D
	18hpf	3048		14		Fig. S4B
	24hpf	5271		19		Fig. S4C
	Overall	13166		45		Fig. 5A, Fig. S3A
Mouse Organogenesis	E9.5 E1S1	5913	25568	12		Fig. S5A

Spatiotemporal Transcriptomic Atlas[37]	E9.5 E2S1	5292	23756	14		Fig. S5B
	E9.5 E2S2	4356	24107	13		Fig. S5C
	E9.5 E2S3	5059	24238			Fig. 6
	E9.5 E2S4	5797	23398			Fig. S5D

Table S4. The summary of clustering performance (ARI scores) on 12 slices of the DLPFC dataset derived by seven methods.

Method	151507	151508	151509	151510	151669	151670	151671	151672	151673	151674	151675	151676	mean
SEDR	0.448	0.407	0.426	0.403	0.295	0.339	0.456	0.503	0.449	0.437	0.460	0.456	0.423
SpaGCN	0.432	0.414	0.417	0.427	0.289	0.360	0.491	0.540	0.397	0.431	0.359	0.309	0.406
STAGATE	0.552	**0.547**	**0.501**	**0.534**	0.262	0.245	0.591	0.571	**0.581**	0.506	**0.602**	0.443	0.494
CCST	0.433	0.302	0.411	0.330	0.342	0.327	0.434	0.434	0.362	0.361	0.367	0.345	0.371
DeepST	0.465	0.253	0.371	0.384	0.658	0.486	0.515	0.608	0.459	0.290	0.401	0.267	0.430
GraphST	0.532	0.462	0.479	0.492	0.390	0.378	0.565	0.588	0.557	0.421	0.589	0.550	0.500
stCluster	**0.645**	0.535	0.477	0.461	**0.733**	**0.503**	**0.746**	**0.682**	0.513	**0.556**	0.548	**0.562**	**0.580**

(Bold numbers represent the best performance for each slice.)

Table S5. The summary of clustering performance (NMI scores) on 12 slices of the DLPFC dataset derived by seven methods.

Method	151507	151508	151509	151510	151669	151670	151671	151672	151673	151674	151675	151676	mean
SEDR	0.581	0.504	0.582	0.519	0.513	0.509	0.585	0.616	0.622	0.560	0.570	0.597	0.563
SpaGCN	0.557	0.519	0.535	0.543	0.397	0.485	0.593	0.600	0.532	0.538	0.436	0.497	0.519
STAGATE	0.662	0.644	**0.637**	0.583	0.510	**0.534**	**0.694**	0.600	**0.709**	0.536	0.571	0.566	0.604
CCST	0.631	0.509	0.604	0.552	0.507	0.463	0.614	0.578	0.537	0.505	0.553	0.537	0.549
DeepST	0.563	0.393	0.507	0.514	0.558	0.514	0.606	0.582	0.542	0.405	0.487	0.392	0.505
GraphST	0.680	0.618	0.635	**0.605**	0.542	0.509	0.661	**0.656**	0.685	0.589	**0.694**	0.641	0.626
stCluster	**0.704**	**0.658**	0.614	0.580	**0.684**	0.527	0.668	0.645	0.629	**0.690**	0.679	**0.690**	**0.647**

(Bold numbers represent the best performance for each slice.)

Table S6 The summary of clustering performance based on the Intersection over Union (IoU) metric on 12 slices of the DLPFC dataset derived by seven methods.

Method	151507	151508	151509	151510	151669	151670	151671	151672	151673	151674	151675	151676	mean
SEDR	0.480	0.429	0.457	0.442	0.355	0.372	0.420	0.514	0.423	0.490	0.433	0.414	0.436
SpaGCN	0.430	0.421	0.450	0.422	0.276	0.383	0.531	0.538	0.406	0.449	0.338	0.339	0.415
STAGATE	0.575	**0.582**	**0.549**	**0.514**	0.247	0.292	0.581	0.563	**0.618**	0.506	**0.596**	0.440	0.505
CCST	0.360	0.324	0.372	0.305	0.334	0.321	0.299	0.315	0.361	0.388	0.333	0.376	0.341
DeepST	0.613	0.425	0.477	0.471	**0.643**	0.471	0.527	**0.609**	0.576	0.429	0.539	0.408	0.516
GraphST	0.482	0.447	0.504	0.411	0.423	0.404	0.587	0.575	0.526	0.437	0.547	0.522	0.489
stCluster	**0.679**	0.570	0.506	0.409	0.568	**0.502**	**0.603**	0.591	0.487	**0.520**	0.518	**0.534**	**0.541**

(Bold numbers represent the best performance for each slice.)

Reference

[1] L. Scrucca, M. Fop, T. B. Murphy, and A. E. Raftery, “mclust 5: Clustering, Classification and Density Estimation Using Gaussian Finite Mixture Models,” *R J.*, vol. 8, no. 1, pp. 289–317, 2016.

[2] J. MacQueen, “Some methods for classification and analysis of multivariate observations,” *Proc. Fifth Berkeley Symp. Math. Stat. Probab. Vol. 1 Stat.*, vol. 5.1, pp. 281–298, Jan. 1967.

[3] U. von Luxburg, “A tutorial on spectral clustering,” *Stat. Comput.*, vol. 17, no. 4, pp. 395–416, Dec. 2007, doi: 10.1007/s11222-007-9033-z.

[4] M. L. Zepeda-Mendoza and O. Resendis-Antonio, “Hierarchical Agglomerative Clustering,” in *Encyclopedia of Systems Biology*, W. Dubitzky, O. Wolkenhauer, K.-H. Cho, and H. Yokota, Eds., New York, NY: Springer, 2013, pp. 886–887. doi: 10.1007/978-1-4419-9863-7_1371.

[5] V. D. Blondel, J.-L. Guillaume, R. Lambiotte, and E. Lefebvre, “Fast unfolding of communities in large networks,” *J. Stat. Mech. Theory Exp.*, vol. 2008, no. 10, p. P10008, Oct. 2008, doi: 10.1088/1742-5468/2008/10/P10008.

[6] P. J. Rousseeuw, “Silhouettes: A graphical aid to the interpretation and validation of cluster analysis,” *J. Comput. Appl. Math.*, vol. 20, pp. 53–65, Nov. 1987, doi: 10.1016/0377-0427(87)90125-7.

[7] E. Fix and J. L. Hodges, “Discriminatory Analysis. Nonparametric Discrimination: Consistency Properties,” *Int. Stat. Rev. Rev. Int. Stat.*, vol. 57, no. 3, p. 238, Dec. 1989, doi: 10.2307/1403797.

[8] J. Hu *et al.*, “SpaGCN: Integrating gene expression, spatial location and histology to identify spatial domains and spatially variable genes by graph convolutional network,” *Nat. Methods*, vol. 18, no. 11, Art. no. 11, Nov. 2021, doi: 10.1038/s41592-021-01255-8.

[9] Y. Long *et al.*, “Spatially informed clustering, integration, and deconvolution of spatial transcriptomics with GraphST,” *Nat. Commun.*, vol. 14, no. 1, Art. no. 1, Mar. 2023, doi: 10.1038/s41467-023-36796-3.

[10] J. Li, S. Chen, X. Pan, Y. Yuan, and H.-B. Shen, “Cell clustering for spatial transcriptomics data with graph neural networks,” *Nat. Comput. Sci.*, vol. 2, no. 6, pp. 399–408, Jun. 2022, doi: 10.1038/s43588-022-00266-5.

[11] K. Dong and S. Zhang, “Deciphering spatial domains from spatially resolved transcriptomics with an adaptive graph attention auto-encoder,” *Nat. Commun.*, vol. 13, no. 1, p. 1739, Apr. 2022, doi: 10.1038/s41467-022-29439-6.

[12] C. Xu *et al.*, “DeepST: identifying spatial domains in spatial transcriptomics by deep learning,” *Nucleic Acids Res.*, vol. 50, no. 22, p. e131, Dec. 2022, doi: 10.1093/nar/gkac901.

[13] G. E. Hinton and R. Zemel, “Autoencoders, Minimum Description Length and Helmholtz Free Energy,” in *Advances in Neural Information Processing Systems*, Morgan-Kaufmann, 1993. Accessed: Feb. 02, 2023. [Online]. Available: https://proceedings.neurips.cc/paper/1993/hash/9e3cfc48eccf81a0d57663e129aef3cb-Abstract.html

[14] D. P. Kingma and M. Welling, “Auto-Encoding Variational Bayes.” arXiv, Dec. 10, 2022. doi: 10.48550/arXiv.1312.6114.

[15] T. N. Kipf and M. Welling, “Semi-Supervised Classification with Graph Convolutional Networks.” arXiv, Feb. 22, 2017. doi: 10.48550/arXiv.1609.02907.

[16] P. Veličković, G. Cucurull, A. Casanova, A. Romero, P. Liò, and Y. Bengio, “Graph Attention

Networks.” arXiv, Feb. 04, 2018. doi: 10.48550/arXiv.1710.10903.

[17] P. Veličković, W. Fedus, W. L. Hamilton, P. Liò, Y. Bengio, and R. D. Hjelm, “Deep Graph Infomax.” arXiv, Dec. 21, 2018. Accessed: Feb. 02, 2023. [Online]. Available: http://arxiv.org/abs/1809.10341

[18] “Mean Squared Error,” in *The Concise Encyclopedia of Statistics*, New York, NY: Springer, 2008, pp. 337–339. doi: 10.1007/978-0-387-32833-1_251.

[19] I. J. Good, “Rational Decisions,” *J. R. Stat. Soc. Ser. B Methodol.*, vol. 14, no. 1, pp. 107–114, 1952.

[20] J. Shlens, “Notes on Kullback-Leibler Divergence and Likelihood.” arXiv, Apr. 07, 2014. doi: 10.48550/arXiv.1404.2000.

[21] V. A. Traag, L. Waltman, and N. J. van Eck, “From Louvain to Leiden: guaranteeing well-connected communities,” *Sci. Rep.*, vol. 9, no. 1, Art. no. 1, Mar. 2019, doi: 10.1038/s41598-019-41695-z.

[22] J. Xie, R. Girshick, and A. Farhadi, “Unsupervised Deep Embedding for Clustering Analysis.” arXiv, May 24, 2016. Accessed: Feb. 01, 2023. [Online]. Available: http://arxiv.org/abs/1511.06335

[23] K. Y. Yeung and W. L. Ruzzo, “Details of the Adjusted Rand indexand Clustering algorithms Supplement to the paper ‘An empirical study on Principal Component Analysis for clustering gene expression data’ (to appear in Bioinformatics)”.

[24] L. Hubert and P. Arabie, “Comparing partitions,” *J. Classif.*, vol. 2, no. 1, pp. 193–218, Dec. 1985, doi: 10.1007/BF01908075.

[25] A. J. Gates and Y.-Y. Ahn, “The Impact of Random Models on Clustering Similarity,” *J. Mach. Learn. Res.*, vol. 18, no. 87, pp. 1–28, 2017.

[26] A. H. Murphy, “The Finley Affair: A Signal Event in the History of Forecast Verification,” *Weather Forecast.*, vol. 11, no. 1, pp. 3–20, Mar. 1996, doi: 10.1175/1520-0434(1996)011<0003:TFAASE>2.0.CO;2.

[27] H. W. Kuhn, “The Hungarian method for the assignment problem,” *Nav. Res. Logist. Q.*, vol. 2, no. 1–2, pp. 83–97, 1955, doi: 10.1002/nav.3800020109.

[28] J. Chen *et al.*, “Unsupervised Spatially Embedded Deep Representation of Spatial Transcriptomics,” In Review, preprint, Jul. 2021. doi: 10.21203/rs.3.rs-665505/v1.

[29] T. Stuart *et al.*, “Comprehensive Integration of Single-Cell Data,” *Cell*, vol. 177, no. 7, pp. 1888-1902.e21, Jun. 2019, doi: 10.1016/j.cell.2019.05.031.

[30] R. Dries *et al.*, “Giotto: a toolbox for integrative analysis and visualization of spatial expression data,” *Genome Biol.*, vol. 22, no. 1, p. 78, Dec. 2021, doi: 10.1186/s13059-021-02286-2.

[31] D. Pham *et al.*, “stLearn: integrating spatial location, tissue morphology and gene expression to find cell types, cell-cell interactions and spatial trajectories within undissociated tissues.” bioRxiv, p. 2020.05.31.125658, May 31, 2020. doi: 10.1101/2020.05.31.125658.

[32] E. Zhao *et al.*, “Spatial transcriptomics at subspot resolution with BayesSpace,” *Nat. Biotechnol.*, vol. 39, no. 11, pp. 1375–1384, Nov. 2021, doi: 10.1038/s41587-021-00935-2.

[33] H. Ren, B. L. Walker, Z. Cang, and Q. Nie, “Identifying multicellular spatiotemporal organization of cells with SpaceFlow,” *Nat. Commun.*, vol. 13, no. 1, Art. no. 1, Jul. 2022, doi: 10.1038/s41467-022-31739-w.

[34] K. R. Maynard *et al.*, “Transcriptome-scale spatial gene expression in the human dorsolateral

prefrontal cortex," *Nat. Neurosci.*, vol. 24, no. 3, Art. no. 3, Mar. 2021, doi: 10.1038/s41593-020-00787-0.

[35] S. Codeluppi *et al.*, "Spatial organization of the somatosensory cortex revealed by osmFISH," *Nat. Methods*, vol. 15, no. 11, Art. no. 11, Nov. 2018, doi: 10.1038/s41592-018-0175-z.

[36] C. Liu *et al.*, "Spatiotemporal mapping of gene expression landscapes and developmental trajectories during zebrafish embryogenesis," *Dev. Cell*, vol. 57, no. 10, pp. 1284-1298.e5, May 2022, doi: 10.1016/j.devcel.2022.04.009.

[37] A. Chen *et al.*, "Spatiotemporal transcriptomic atlas of mouse organogenesis using DNA nanoball-patterned arrays," *Cell*, vol. 185, no. 10, pp. 1777-1792.e21, May 2022, doi: 10.1016/j.cell.2022.04.003.
